# A novel afterglow nanoreporter for monitoring cancer therapy

**DOI:** 10.7150/thno.77457

**Published:** 2022-09-25

**Authors:** Shiyi Liao, Youjuan Wang, Zhe Li, Ying Zhang, Xia Yin, Shuangyan Huan, Xiao-Bing Zhang, Sulai Liu, Guosheng Song

**Affiliations:** 1 State Key Laboratory of Chemo/Bio-Sensing and Chemometrics, College of Chemistry and Chemical Engineering, Hunan University, Changsha 410082, P. R. China.; 2 Department of Hepatobiliary Surgery, Hunan Provincial People's Hospital/The First Affiliated Hospital of Hunan Normal University, Changsha, Hunan Province, People's Republic of China.

**Keywords:** Molecular engineering, Semiconducting polymer nanoparticle, Afterglow imaging, Immunogenic cell death, Therapy monitoring

## Abstract

**Rationale:** Immunogenic cell death (ICD)-associated immunogenicity evoked through reactive oxygen species (ROS) is an efficient way to fight against the immune-dysfunctional microenvironment, so as to provoke potent anti-tumor immunity. However, the unknown ROS dose during cancer therapies may induce adverse immune responses (e.g., insufficient ICD, toxicity toward normal tissues or immune system).

**Methods:** Herein, we developed a pyrido pyrazine - thiophene based semiconducting polymer as novel near-infrared (NIR) organic afterglow nanoparticles for the real-time visualization of self-generated ROS, during photodynamic-mediated immunogenic cell death. Specifically, we introduced the strong “acceptor” (pyrido pyrazine) into thiophene based semiconducting polymer to redshift emission wavelength, and further modulate the “donor” to afford more afterglow reaction sites and reducing ΔEst, so as to enhance luminescence intensity.

**Results:** The semiconducting polymer-based afterglow nanoparticles exhibit strong afterglow emission with longer-wavelength emission (> 800 nm), compared with the reported organic afterglow nanoparticles (e.g., MEHPPV, PFODBT or Chlorin, < 690 nm), which endows this afterglow nanoparticles with a greatly improvement of signal to noise ratio. Moreover, the photodynamic effect of this afterglow nanoparticles can induce immunogenic cell death of cancer cells and further cause immune responses in mice.

**Conclusions:** The NIR afterglow signal presents a good relationship with ROS generation, immunogenic cell death and outcome of treatment. Therefore, it was able to provide a non-invasive tool for predicting the degree of ICD that occurs during ROS-mediated cancer therapy and may contribute to precise immunotherapy.

## Introduction

Immunotherapy that boosts the natural defenses of body to fight against cancer has emerged as a new method of cancer treatment 1, 2. Despite some recent clinical success using cancer immunotherapy, only a small portion of patients with certain types of cancers benefit from this treatment. One of the main reasons is that cancer cells featured with a high mutation rate can produce *de novo* antigens, which leads to low immunogenicity and poor recognition by dendritic cells (DCs), thus evasion from the immune response. To promote the immune response, a facile approach is to induce the immunogenic cell death (ICD) of tumor cells 3, 4. Photodynamic therapy (PDT), which use the reactive oxygen species (ROS) produced from the molecular oxygen in the presence of photosensitizers and suitable excitation lights, is recently emerged as complementary and unconventional oncology treatment and may be a suitable palliative treatment option as it is a minimally invasive procedure 5-8. ROS produced in the process of PDT can cause apoptosis of tumor cells 9, accompanied by the release of damage-associated molecular patterns (DAMPs) including adenosine triphosphate (ATP) or calreticulin (CRT), and high mobility group box 1 (HMGB1) from dying tumor cells 10-13. These DAMPs then cause the maturation of DCs and the activation of effector T cells, increasing the host antitumor immune response 14-18. However, the uncertain ROS dose during treatment is a limiting factor leading to the uncertainty of anti-cancer efficacy 19, 20. On one hand, the low dosage of ROS may induce not enough DAMPs to cause sufficient ICD to kill tumors 21. On the other hand, an excessive amount of ROS could cause harm to nearby normal tissue or immune cells. As a result, real-time monitoring of ROS generation is critical for precise photodynamic therapy and ROS mediated ICD.

Optical imaging has extreme significance in the prevention and treatment of malignant cancer for nearly half a century 22-24. With no need for real-time excitation, afterglow nanoparticles can efficiently eliminate the background signals from autofluorescence of bio-tissues, overcoming the common drawback of fluorescence imaging 25, 26. Due to the significant enhancement of signal-to-background ratio and imaging quality, afterglow luminescence has been broadly used for *in vivo* imaging, including cell tracking, bio-detection and lymph node location with a non-invasive way 27-31. Since 1996, a variety of inorganic afterglow nanoparticles have been reported. However, we cannot ignore the toxicity of inorganic materials when they are used in living body 32-34. This is because inorganic afterglow nanoparticles usually contain rare-earth heavy metals such as Gd, Yb and Eu. As a comparison, organic afterglow luminescence nanoparticles that are biocompatibility have been attracted more and more attention in recent years 35-38. Even though, there are still limited candidates of organic afterglow. Moreover, the reported organic afterglow materials (such as MEHPPV, PFODBT or Chlorin) are still hampered in real practice by the relatively short emission wavelength (less than 595 nm, 690 nm, or 670 nm, respectively) 23, 39, 40. Because the extension of molecular emission wavelength will bring about the reduction of photoluminescence quantum yield due to the energy gap law, it is difficult to synthesize near-infrared (NIR) afterglow candidates together with strong emission intensity 41. Besides, due to the insufficient spectral overlap between donors and acceptors, it remains a difficult task using fluorescence resonance energy transfer (FRET) to prepare highly efficient afterglow materials with long λ_em_ 42-44. Therefore, it is necessary to design novel afterglow nanoparticle with NIR emission.

Herein, we introduce pyrido pyrazine (PP) into thiophene based semiconducting polymer to design a novel afterglow candidate with near-infrared (NIR) emission for afterglow imaging of self-generated ROS during ROS-mediated cancer immunotherapy. Specifically, we designed three kinds of donor-acceptor conjugated (D-A) copolymers, during which the enhanced internal charge transfer between the “donor” and the “acceptor” moieties resulted in the red-shifting of the wavelength. Moreover, we introduced more thiophene groups as reaction sites for reducing ΔEst and driving the afterglow luminescence. Through a series of molecular structure engineering, NIR-3 based afterglow nanoparticles SPN (NIR-3) were obtained owning near-infrared afterglow emission itself without the need of multi-components to red-shift the emission wavelength. Importantly, singlet oxygen (^1^O_2_) generated from SPN(NIR-3) not only participated into the afterglow emitting process, but also was capable of inducing effective ICD, which was accompanied with the production of DAMPs, boosting cancer cell antigenicity. Furthermore, afterglow signals were correlated well with ^1^O_2_ generation, ICD levels, and cancer inhibition rates. Finally, such an afterglow nanoreporter provided facile and alternative parameters for predicting ^1^O_2_-mediated ICD, immune response and treatment outcome during immunotherapy.

## Results

### Design, synthesis, and characterization of near-infrared afterglow semiconducting polymers

**Figure 1:**
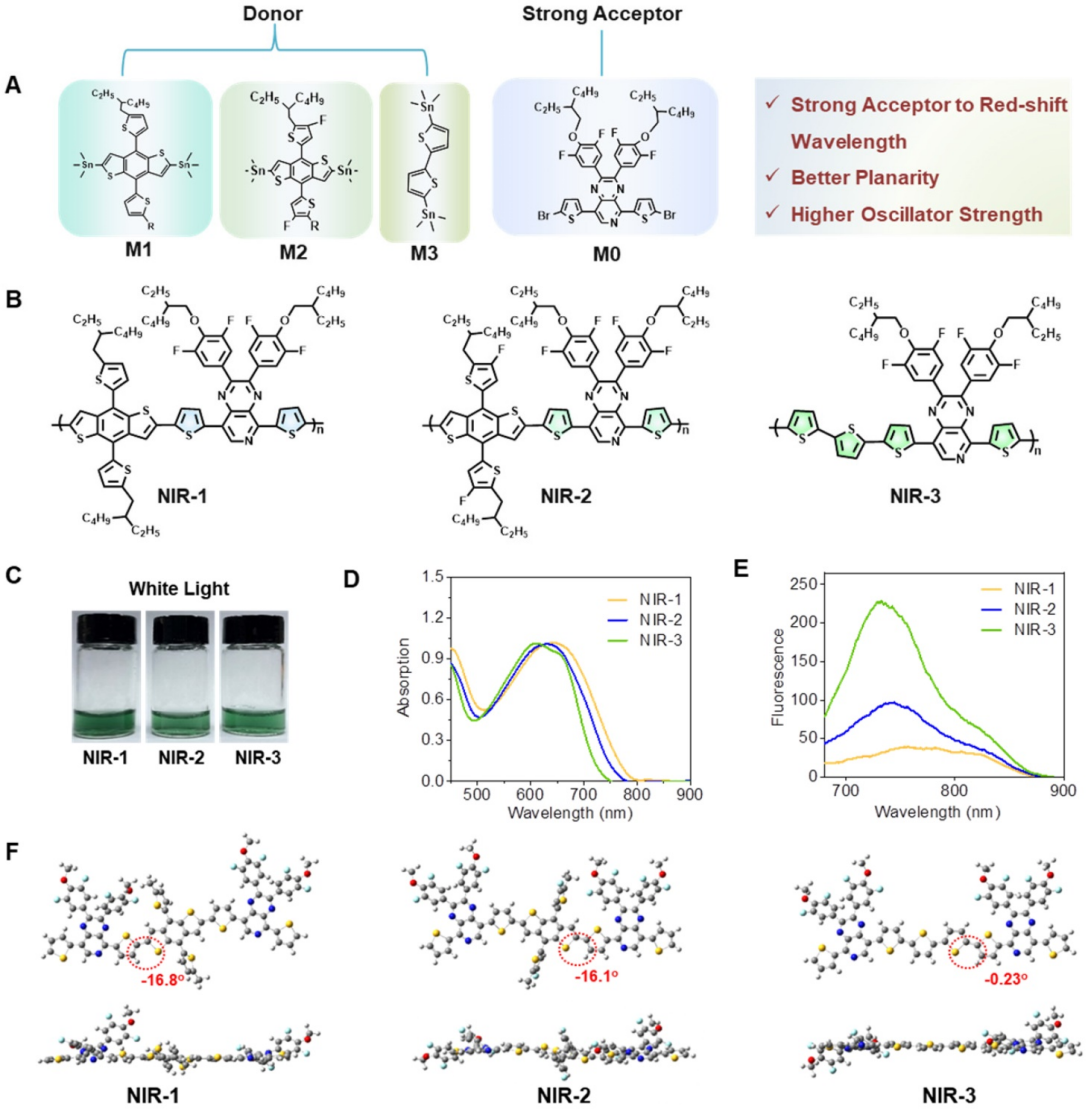
** Design, Synthesis, and Characterization of Near-Infrared Afterglow Semiconducting Polymers. (A)** Illustration of molecular of three SPs. **(B)** Molecular structure of three SPs. **(C)** Photographs of three SPs. **(D)** UV-Vis spectra of various SPs. **(E)** Fluorescence spectra of various SPs. **(F)** The dihedral angles for three SPs by density functional theory. Exchange functional: B3LYP. Basis sets: 6-31G*.

To develop new organic afterglow agents, we synthesized three kinds of semiconducting polymers (SPs). The monomer of 5,8-bis(5-bromothiophen-2-yl)-2,3-bis(4-((2-ethylhexyl) oxy)-3,5-difluorophenyl) pyrido[3,4-b] pyrazine (M0) was synthesized following the previous 45. And then, the corresponding polymers were obtained by copolymerization of monomers with different trimethylstannane. The polymer NIR-1 was synthesized by a stille coupling reaction between M0 and 4,8-bis (5-(2-ethylhexyl) thiophen-2-yl) benzo[1,2-b:4,5-b'] dithiophene-2,6-diyl) bis (trimethylstannane) (M1). Difluoro-substituted-M1 (M2) and 5,5-Ditrimethylstannyl-2,2'-bithiophene (M3) were selected to obtain NIR-2 and NIR-3, respectively (Figure 1A-C, S1) 45. Next, NIR-1/NIR-2/NIR-3 were characterazed by ^1^H NMR spectra and GPC measurement (Figure S2-5). Figure 1c showed the photograph of three SPs in tetrahydrofuran (THF). The UV-Vis and fluorescence spectra of the molecules demonstrated that NIR-1/NIR-2/NIR-3 had absorption at 640 nm and had near-infrared (NIR) emission properties (Figure 1D, E), indicating their NIR afterglow emission potential. Moreover, the optimized geometric structures of NIR-1/NIR-2/NIR-3 were studied by density functional theory (DFT) with the Gauss 09 program at the B3LYP/6-31G* level. The dihedral angle between thiophene and pyrido pyrazine plane in NIR-3 was -0.23°, which was much smaller than in NIR-1(-16.8°) and NIR-2(-16.1°) (Figure 1F). This optimized geometric structure data revealed that NIR-3 possesses a relatively more coplanarity than NIR-1 and NIR-2. Furthermore, the biggest oscillator strength was consistent with the enhanced fluorescence intensity of NIR-3 (Figure S6) 46. As depicted in Figure S7, for both NIR-1, NIR-2 and NIR-3, the highest occupied molecular orbital (HOMO) mainly localizes on the skeleton of molecules, whereas the lowest unoccupied molecular orbital (LUMO) mostly distributes on the electron-rich donor moiety.

### Screening of semiconducting polymer nanoparticles for near-infrared afterglow luminescence

**Figure 2:**
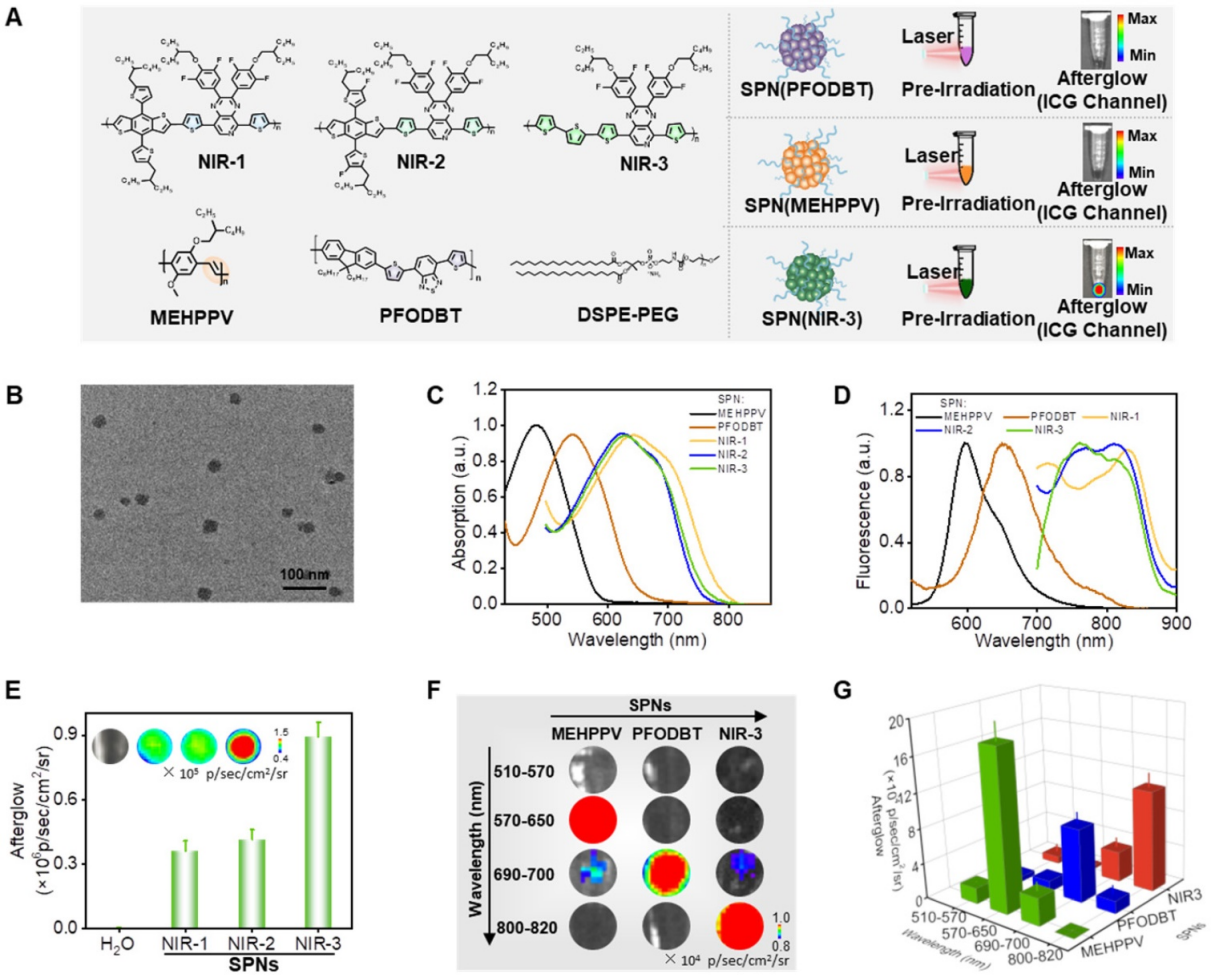
** Screening of Near-Infrared Afterglow Nano-Reporter. (A)** Molecular structures of various afterglow molecules and the illustration of the preparation of SPN *via* nanoprecipitation. **(B)** Transmission electron microscope image of SPN(NIR-3). **(C)** Absorption spectra of various SPN. **(D)** Fluorescence spectra of various SPN. **(E)** Afterglow intensities of SPN(NIR-1/NIR-2/NIR-3) (The inset is the afterglow image). **(F)** Afterglow emission of various SPN, obtained through different channels (GFP: 510-570 nm; DsRed: 570-650 nm; Cy5.5: 690-770 nm; ICG: 820-880 nm). **(G)** The corresponding afterglow intensity, quantified from (F).

MEHPPV and PFODBT have been shown to be effective organic afterglow materials in the past research, so the five SPs were then self-assembled into semiconducting polymer nanoparticles (SPN) *via* nanoprecipitation using amphiphilic copolymers (1, 2-dimyristoyl-sn-glycero-3-phosphoethanolamine-N- [methoxy (polyethylene glycol) (DSPE-PEG, Mw=2000) as surfactants, respectively (Figure 2A). Next, we systematically characterized the dynamic light scattering (DLS), transmission electron microscope (TEM), UV-Vis absorption spectra and fluorescence spectra of these SPN. Typically, the SPN(NIR-3) had a spherical morphology with an average diameter of 30 nm, as demonstrated by TEM images (Figure 2B). The DLS showed that the size of these SPN were ranging from 35 nm to 70 nm (Figure S8). SPN(MEHPPV) and SPN(PFODBT) showed UV-Vis absorption peaks at 495 and 550 nm, respectively. Meanwhile, SPN(NIR-1/NIR-2/NIR-3) had similar absorption spectra with a maximum peak at 635 nm (Figure 2C). As shown in figure 2D, SPN(NIR-1/NIR-2/NIR-3) displayed the wide fluorescence emission from 800-850 nm, which was a red-shift of 230 nm and 175 nm over SPN(MEHPPV) and SPN(PFODBT), respectively. Following that, we used the IVIS living animal imaging system to gather the afterglow signals of SPN(NIR-1/NIR-2/NIR-3) in bioluminescence modes. SPN(NIR-3) exhibited the strongest afterglow luminescence among SPN(NIR-1), SPN(NIR-2) and SPN(NIR-3) and thus was selected for further comparison (Figure 2E). Then, we used four different channels (GFP: 510-570 nm; DsRed: 570-650 nm; Cy5.5: 690-770 nm; ICG: 820-880 nm) to collect afterglow signals of SPN(NIR-3), SPN(MEHPPV) and SPN(PFODBT). The afterglow luminescence emission of SPN(MEHPPV) and SPN(PFODBT) were mainly observed from the DsRed and Cy5.5 channel, respectively. Whereas, the afterglow luminescence for SPN(NIR-3) was mainly concentrated in ICG channel, enabling NIR afterglow imaging capability (Figure 2F, G).

### Investigation of mechanism for near-infrared afterglow luminescence

**Figure 3:**
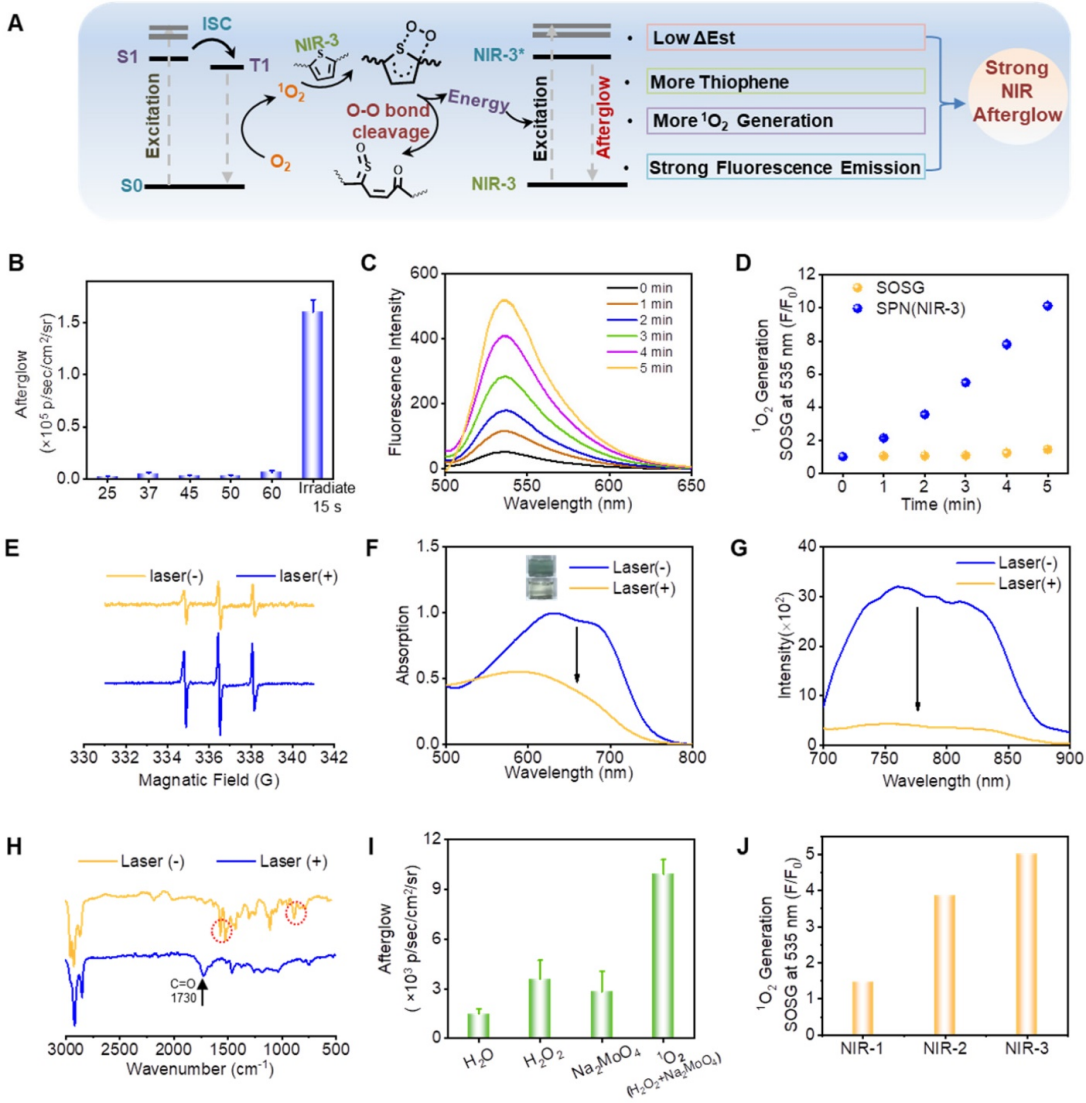
**Investigation of Mechanism for Near-Infrared Afterglow Luminescence. (A)** The illustration of afterglow luminescence mechanism. **(B)** Afterglow intensities of SPN(NIR-3) excited by heating at various temperatures (e.g., 25, 37, 45, and 60 °C) or 660 nm laser irradiation (15 s). **(C)** Fluorescence of SOSG mixed with SPN(NIR-3) after receiving different irradiation times. **(D)** Fluorescence enhancement (F/F_0_) of SOSG at 535 nm for SPN(NIR-3) after 660 nm laser irradiation. **(E)** ESR spectra of TEMP + SPN(NIR-3) with or without irradiation. **(F)** The absorption spectra of NIR-3 before and after irradiation. **(G)** The fluorescence spectra of NIR-3 before and after irradiation. **(H)** FITR spectra of NIR-3 before and after irradiation. **(I)** Luminescent intensity of SPN(NIR-3) incubated with PBS, Na_2_MoO_4_, H_2_O_2_ or Na_2_MoO_4_ + H_2_O_2_. **(J)** ^1^O_2_ generation capacity of SPN (NIR-1/NIR-2/NIR-3).

The underlying mechanism for brightest near-infrared (NIR) afterglow of SPN(NIR-3) were then investigated. First, we looked into the effect of temperature on afterglow luminescence, and discovered that even when SPN(NIR-3) was heated to 60 °C, there was no discernible afterglow luminescence. After SPN(NIR-3) was subjected to 15 seconds of 660 nm laser irradiation (0.8 W/cm^2^), a significant NIR afterglow signal was seen (Figure 3B). The production of ^1^O_2_ from SPN(NIR-3) was detected using SOSG. The fluorescent intensity of SOSG at 535 nm rose by a factor of 10 after laser irradiation (Figure 3C, D). Besides, the ESR signal further confirmed the generation of ^1^O_2_ by NIR-3 after irradiation (Figure 3E). By comparing the relationship between the chemical structures of the three different semiconducting polymers (SPs) and their afterglow intensities, we hypothesized that one of the key reaction sites in the afterglow luminescence process was thiophene in SPs. Thus, we investigated the changes in the chemical structure of NIR-3 allowed by laser irradiation. After 4 h irradiation with laser, both absorption and emission peak of SPN(NIR-1/2/3) disappeared, suggesting the decomposition of SPN(NIR-1/2/3) (Figure 3F-G, S9). After irradiation, fourier transform infrared spectroscopy (FTIR) analysis revealed the emergence of a carbonyl peak at 1730 cm^-1^, as well as the disappearance of typical thiophene peaks at 1514 cm^-1^, 1465 cm^-1^ (stretching modes of C=C and C-C in the thiophene ring) and 800 cm^-1^ (C-H out-of-plane vibration within the thiophene ring) after irradiation 47, 48, showing the oxidation of thiophene groups (Figure 3H). Then, we have collected afterglow signals of SPN(NIR-3) under hypoxia or normoxia condition (Figure S10). Afterglow luminescence in normoxia was twice as high as in hypoxia, indicating the necessity of oxygen in the production of afterglow. To explore whether the afterglow luminescence of SPN(NIR-3) could be directly triggered by ROS, SPN(NIR-3) was directly incubated with different ROS, including ^1^O_2_ (produced from the reaction between H_2_O_2_ and Na_2_MoO_4_), H_2_O_2_, O_2_^•-^ and •OH (produced from the reaction between H_2_O_2_ and Fe^2+^). As shown in Figure 3I and S11, the luminous intensity of SPN(NIR-3) excited by Na_2_MoO_4_ + H_2_O_2_ was much higher than other ROS. Meanwhile, The ESR spectra showed no obvious O_2_^•-^ and •OH production after irradiation (Figure S12). These findings suggested the following mechanism for the afterglow of SPs taking NIR-3 as an example, i.e., NIR-3 passed the absorbed energy to oxygen to produce ^1^O_2_, which subsequently oxidized the thiophene group to form a thiophene-dioxetane intermediate 39, and the decomposition of the thiophene-dioxetane intermediate finally led to the afterglow luminescence (Figure 3A).

According to the above mechanism, we speculated one of the reasons why NIR-3 had the brightest afterglow luminescence was related to the largest number of thiophene structures in its main chain. Since the afterglow luminescence process was related to ^1^O_2_,^ 1^O_2_ generation capabilities of the those molecules or nanoparticles were investigated using SOSG as the ^1^O_2_ indicator. Thereby, another possible reason is that SPN(NIR-3) produced the largest generation efficiency of ^1^O_2_ after laser irradiation (Figure 3J and S13). As shown in Figure S6, NIR-3 displayed lowest ΔEst among NIR-1, NIR-2 and NIR-3, indicating that NIR-3 was more likely to undergo intersystem crossing (ISC) and then produced more ^1^O_2_ 49. Besides, the strongest fluorescence emission intensity of NIR-3 might also contribute to the strongest afterglow luminescence of SPN(NIR-3).

### Systematic study of near-infrared afterglow properties for SPN(NIR-3)

**Figure 4:**
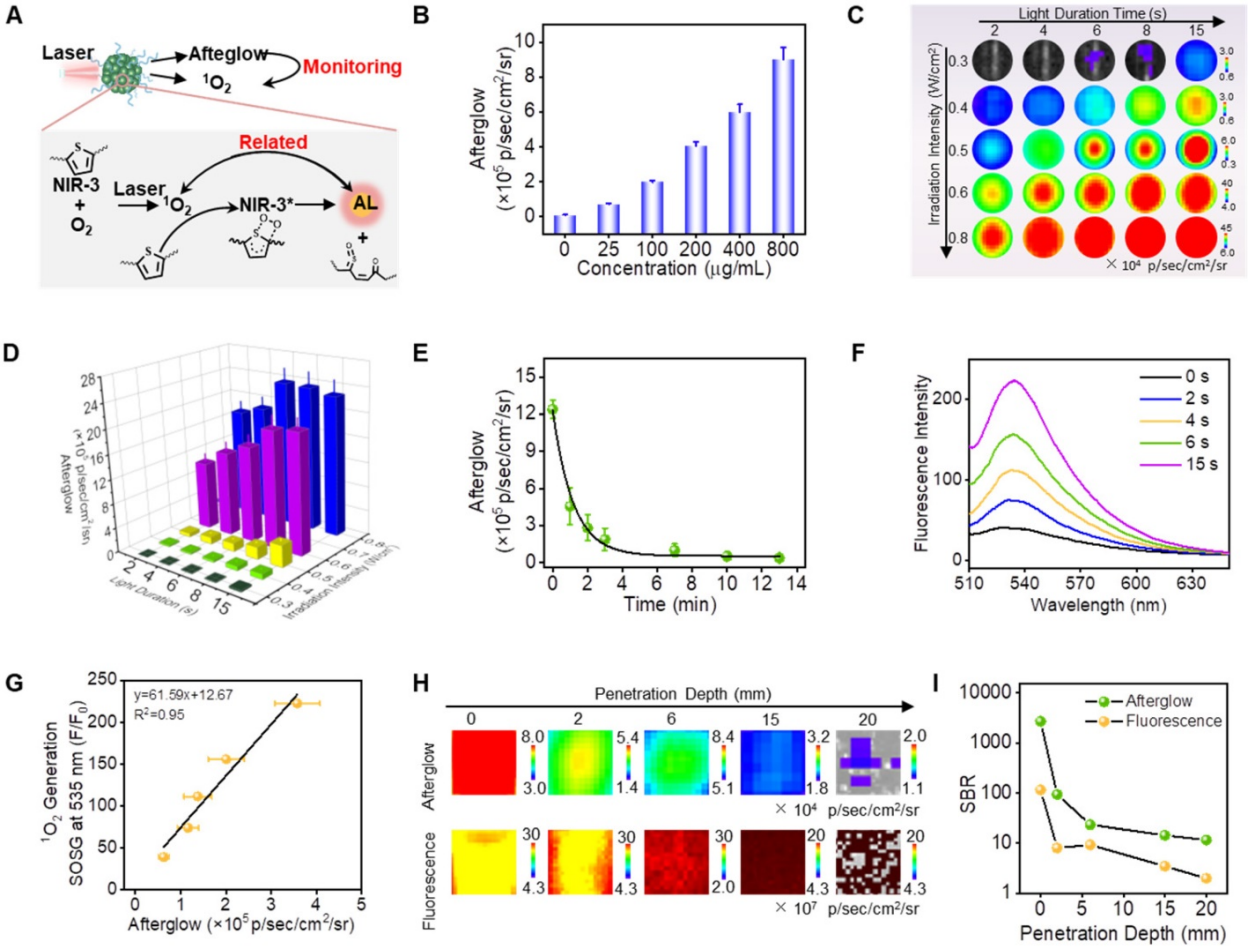
** Systematic Study of Near-Infrared Afterglow Properties for SPN(NIR-3). (A)** Illustration of the correlation between afterglow intensity and singlet oxygen production. **(B)** Afterglow intensities of SPN(NIR-3) with different concentrations of SPN(NIR-3). **(C)** Afterglow images of SPN(NIR-3) with different excitation power and different irradiation times. **(D)** The corresponding afterglow intensity, quantified from (C). **(E)** Luminescent decay study of SPN(NIR-3). **(F)** Fluorescence spectra of SOSG: ^1^O_2_ generation capacity SPN(NIR-3) with different times irradiation. **(G)** Correlation between afterglow intensity and ^1^O_2_ yield for SPN(NIR-3), calculated from (D) and (F). **(H)** Afterglow luminescence (upper) and fluorescence (lower) images of SPN(NIR-3) through chicken tissues of different thickness. **(I)** Signal-to-background ratio for afterglow luminescence and fluorescence from (G).

Afterwards, to test afterglow luminescence properties of SPN(NIR-3), a comprehensive examination was conducted. Firstly, the afterglow signal of SPN(NIR-3) was increased with the increasing concentration of nanoparticles, which indicated that there was concentration-dependent emission (Figure 4B). The afterglow intensity of SPN(NIR-3) was increased significantly as the power increasing and the irradiation time extending (Figure 4C, D). Meanwhile, SPN(NIR-3) demonstrated continuous brightness for more than 10 minutes (halif-life was 51.6 s) after irradiation ended, as shown in afterglow images (Figure 4E). Moreover, after multiple repetitions of irradiation, the afterglow signals of SPN(NIR-3) could be re-activated (Figure S14). And SPN(NIR-3) showed no obvious change of DLS size after irradiation (Figure S15). Notably, SPN(NIR-3) presented the similar afterglow luminescence in different buffers (H_2_O, PBS (pH=7.4, 6.4 and 5.4) or 1640 cell culture medium) (Figure S16). Furthermore, after preservation in various mediums, no obvious precipitation or size changes were observed (Figure S17), indicating a good colloid stability. Then, the production of ^1^O_2_ from SPN(NIR-3) was then detected using SOSG (Figure 4F). From the underlying mechanism depicted in Figure 4A, we supposed that there was a positive relationship between the ^1^O_2_ yield and the afterglow intensity of SPN(NIR-3). Therefore, we plotted the correlation diagram between these two sets of data, obtained a good connection between the yield of ^1^O_2_ and the NIR afterglow intensities (R^2^=0.95) (Figure 4G).

What's more, we explored signal to noise ratio (SNR) of afterglow luminescence for SPN(NIR-3) under chicken tissue. Notably, even after covering 2 cm of fresh chicken tissue, we were still able to detect clear afterglow sginal, while the autofluorescence from chicken tissue made SPN(NIR-3) indistinguishable in fluorescent images (Figure 4H). Because the background noise for NIR afterglow luminescence (8.022 × 10^2^ psec^-1^cm^-2^sr^-1^) was remarkably lower than that of fluorescence (6.712 × 10^7^ psec^-1^cm^-2^sr^-1^), the SNR for afterglow images of SPN(NIR-3) after covering a 2 cm chicken tissue was 5.8 times than that of fluorescence (Figure 4I). Moreover, when covering 0.2 cm chicken tissue, the SNR of afterglow was even up to 12.3 times higher than that of fluorescence. Meanwhile, the SNR of fluorenscence for SPN(NIR-3) and SPN(MEHPPV) was also explored using mimic tissue. As was shown in Figure S18, fluorescence imaging with longer emssion wavelength could significantly improves SNR in imaging.

### Afterglow luminescence monitoring ^1^O_2_ and correlating with anti-cancer efficiency *in vitro*

**Figure 5:**
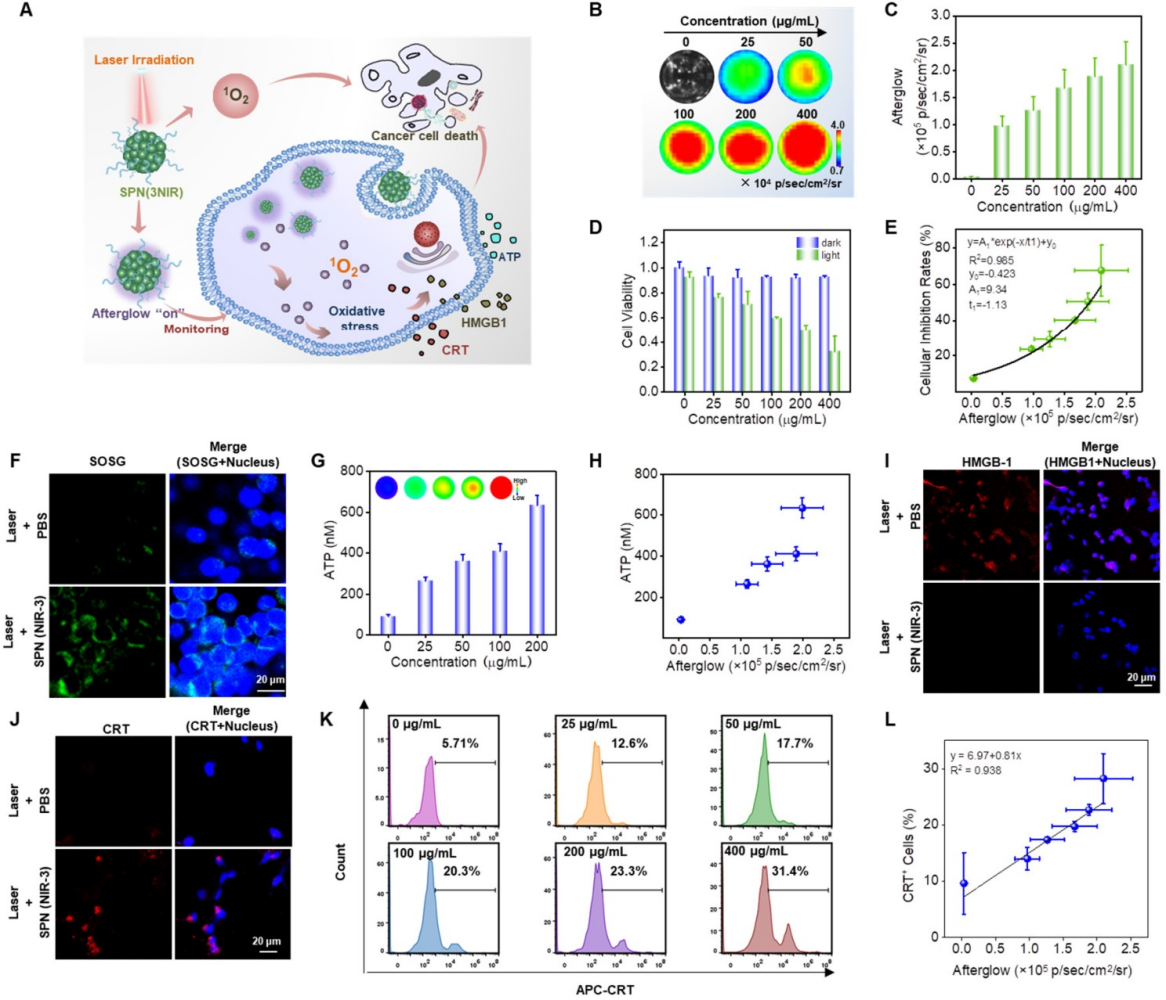
** Afterglow Luminescence Monitored ^1^O_2_ and Correlated with Immunogenic Cell Death and Anti-Cancer Efficiency *In vitro*. (A)** Illustration of the afterglow monitoring photodynamic therapy. **(B)** Afterglow images of CT26 cancer cells incubated with different concentrations of SPN(NIR-3). **(C)** The corresponding afterglow intensity, quantified from (B). **(D)** The relative viability of CT26 cancer cells incubated with different concentrations of SPN(NIR-3), with 660 nm laser irradiation for 10 mins or not. **(E)** Correlation between afterglow intensity and cellular inhibition rate for SPN(NIR-3), calculated from (C) and (D). **(F)** Fluorescent confocal images of CT26 cancer cells treated with SPN(NIR-3) and 660 nm laser irradiation or not. The cell nuclei and ^1^O_2_ were detected using Hochest and SOSG, respectively. **(G)** Quantification of extracellular ATP concentration. **(H)** Correlation between afterglow intensity and the secreted ATP concentration, calculated using data from (C) and (G). **(I-J)** Fluorescent confocal images of CT26 cancer cells incubated with SPN(NIR-3) and 660 nm laser irradiation or not. The cell nuclei, CRT and HMGB1 were detected using Hochest, APC-conjugated anti-CRT antibody, and Alexa Fluor 594-conjugated anti-HMGB1 antibody respectively. **(K)** Flow cytometry analysis of CRT exposure for CT26 cancer cells treated with different concentrations of SPN(NIR-3) + irradiation. **(L)** Correlation between afterglow intensities and normalized CRT levels, calculated using data presented in (C) and (K).

We assumed that the near-infrared (NIR) afterglow luminescence of SPN(NIR-3) could report the self-generated ^1^O_2_ in real time beacause of the positive correlation between NIR afterglow luminescence and ^1^O_2_ generation, which could further predict the cell killing efficiency of SPN(NIR-3) itself (Figure 5A). First, we tested the photodynamic therapy of SPN(NIR-3) toward cancer cells by incubating CT26 cancer cells with different concentrations of SPN(NIR-3) and irradiating them with 660 nm laser (0.6 W/cm^2^). The afterglow signals from those cancer cells were collected using IVIS living animal imaging system and the cellular viability of SPN(NIR-3) was tested by MTT assay.

As the concentration of SPN(NIR-3) increasing, the intensity of those cells' afterglow signal became stronger, while their cellular viability were reduced (Figure 5B-D). Calculated from Figure 5c and 5d, we obtained a good exponential relationship between cell inhibition and afterglow intensity (R^2^=0.988) (Figure 5E). Furthermore, fluorescent confocal images revealed that SPN(NIR-3) during laser irradiation generated a considerable amount of ^1^O_2_ within cancer cells (Figure 5F). Therefore, the success of SPN(NIR-3) as an afterglow imaging-based nanoreporter for real-time monitoring of ^1^O_2_ dose *via* NIR afterglow during cancer photodynamic therapy was highlighted by the findings.

### Photodynamic therapy-elicited immunogenic cell death in cancer cells

Immunogenic cell death (ICD) is an unique way of cell death along with the release of DAMPs such as ATP, CRT, and HMGB1. Among those, the release of ATP stimulated the recruitment of DCs into tumors, CRT increased the engulfment of tumor antigens by DCs, and HMGB1 inspired excellent antigen presentation to T cells during ICD-related immune responses, inducing an effective cytokines-mediated immune response 12, 50, 51. We hypothesized that SPN(NIR-3), as a good photosensitizer, would increase tumor immunogenicity *via* ^1^O_2_-induced ICD. Specifically, the release of three DAMP markers (ATP, CRT, and HMGB1) were used as universal indications to show that SPN(NIR-3) could cause ICD (Figure 5A). To begin with, CT26 cancer cells were treated with various doses of SPN(NIR-3). We used the ATP assay kit and bioluminescent imaging to evaluate ATP levels released from CT26 cancer cells treated with SPN(NIR-3) and laser irradiation according to standard protocols, and it was favorably connected with the concentration of SPN(NIR-3) (Figure 5G). Furthermore, in cancer cells treated with SPN(NIR-3), we discovered a substantial connection (R^2^=0.89) between near-infrared (NIR) afterglow intensity and accreted ATP concentration (Figure 5H). Then, translocation of CRT to cell surface and release of HMGB1 induced by SPN(NIR-3) was imaged using immunofluorescence staining. SPN(NIR-3) with laser irradiation showed fewer HMGB1 and greater CRT signals, as revealed in fluorescent confocal images in Figure 5I, J. Finally, the expression of CRT was measured by flow cytometry after irradiation. We discovered that as the dose of SPN(NIR-3) was increased, CRT expression was steadily elevated, demonstrating that SPN(NIR-3) induced CRT expression is dose-dependent (Figure 5K). Importantly, there were strong postive relationships between the NIR afterglow intensity of SPN(NIR-3) treated cells and CRT level (R^2^=0.938) (Figure 5L). It was reasonable to conclude that SPN(NIR-3) could enhance intracellular oxidative stress by generating ^1^O_2_ under irradiation and was an excellent photosensitizer capable of inducing ICD. Meanwhile, NIR afterglow signals of SPN(NIR-3) were well linked with the ICD levels caused by SPN(NIR-3), indicating that it might be used as an afterglow nano-reporter.

### *In vivo* afterglow luminescence imaging monitoring therapy efficiency

**Figure 6:**
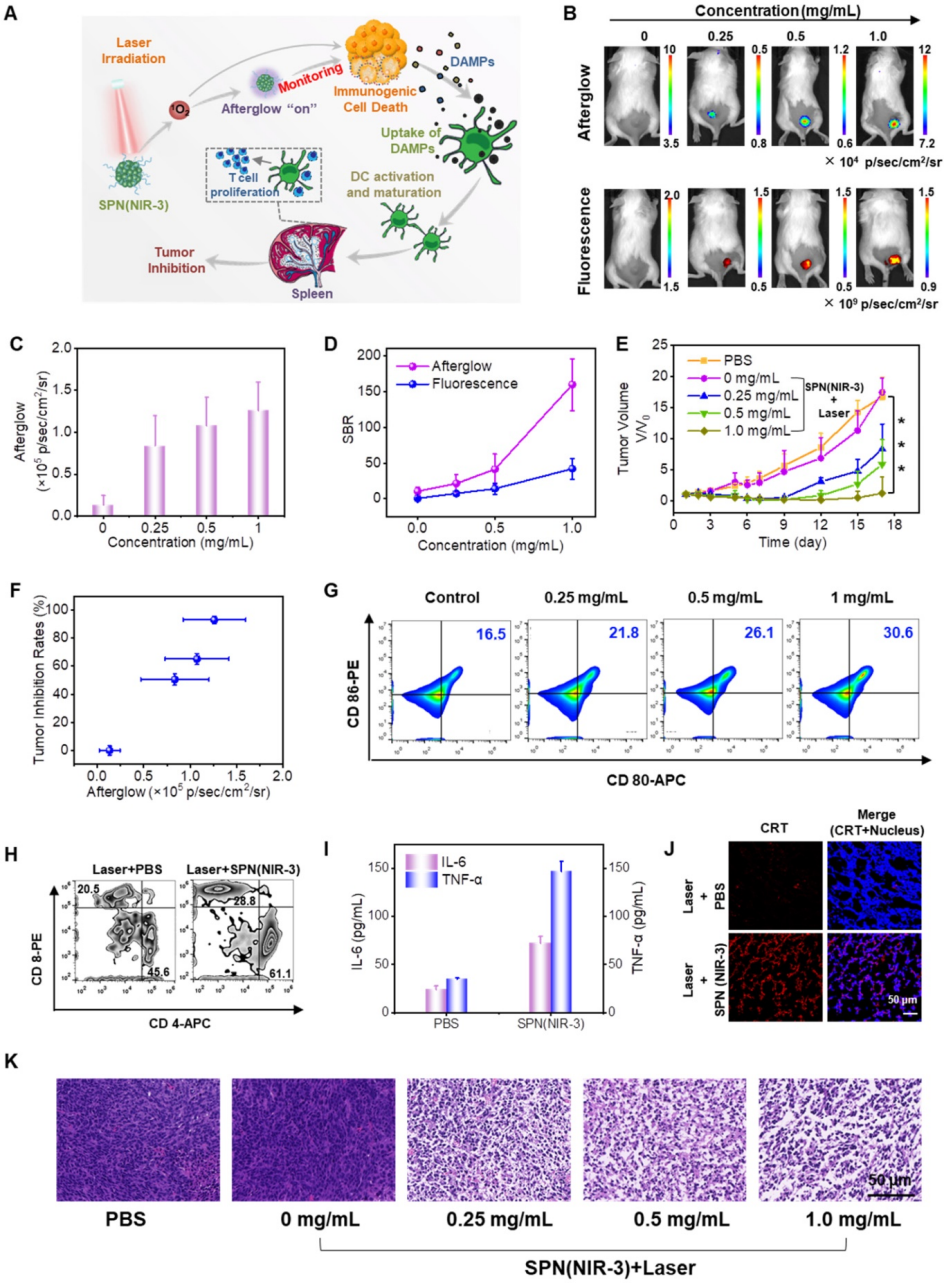
***In vivo* Afterglow Luminescence Imaging Monitoring Immunogenic Cell Death and Therapy Efficiency. (A)** Illustration of ICD and anticancer effect induced by the photodynamic effect of SPN(NIR-3). **(B)** Afterglow (upper panel) and fluorescence (lower panel) images of mice bearing subcutaneous CT26 xenograft tumors i.t. injected with different concentrations of SPN(NIR-3). **(C)** Corresponding quantification of afterglow intensities for tumor areas in (B). **(D)** The signal-to-background ratio for afterglow and fluorescence images. **(E)** Tumor growth curves for each group. **(F)** Correlation between afterglow intensities and tumor growth inhibition rates on the 16th day, calculated using data from (C) and (E). **(G)** Flow cytometry assay of matured DCs (CD80^+^CD86^+^ gated on CD11c^+^) in spleens of mice at the third day post different treatments. **(H)** Populations of CD4^+^ and CD8^+^ T cells in spleens of mice at the fifth days post different treatments. **(I)** Interleukin-6 (IL-6) (purple column) and tumor necrosis factor-α (TNF-α) (blue column) levels in spleens of mice detected using the enzyme-linked immunosorbent assay. **(J)** Representative immunostaining fluorescent confocal images of CRT expression in CT26 tumor slices. **(K)** H&E staining images of tumor slices at the first day post treatment. P-values were determined using one-way ANOVA (*P < 0.05, **P < 0.01, and ***P < 0.001).

To assess the NIR afterglow luminescence imaging performance of SPN(NIR-3) *in vivo*, the afterglow signal of mice bearing subcutaneous CT26 xenograft tumor was recorded after intratumoral (i.t.) injection with different dose of SPN(NIR-3) and then exposed to 660 nm laser irradiation. With increasing injection dose, mice showed the enhanced afterglow signals in the tumor region (Figure 6B). Notably, the tumor displayed a substantial afterglow contrast, but the other parts of mice showed no significant afterglow signal. After quantification of the afterglow and fluorescence images (Figure 6C and S19), the signal to noise ratio in the tumor regions for afterglow was 4-fold higher than that for fluorescence (Figure 6D).

Subsequently, those tumors received another irradiation for an additional 660 nm laser (total 8 minutes, 0.8 W/cm^2^) to impose photodynamic effect toward to tumor. Notably, there was a six-minute interval after the end of every two minutes of irradiation to prevent the tumor area from overheating. Every other day, the tumor volumes was measured to determine the tumor inhibition rate following treatment. The tumor growth curves revealed that as the injection of SPN(NIR-3) dose rising, the tumor inhibition rate was increased considerably (Figure 6E), suggesting remarkable therapeutic efficacy. Furthermore, the afterglow intensity of tumor regions was correlated with tumor growth inhibition. Specifically, those tumors that showed higher NIR afterglow signal were significantly regressed, while those tumor that exhibited the low afterglow signal were negligibly inhibited (Figure 6F). And the tumor necrosis was validated by hematoxylin and eosin (H&E) staining of tumor slices, which matched the tumor growth curves (Figure 6K). These results presented a good relationship between NIR afterglow luminescence intensities of SPN(NIR-3) and anticancer efficiency. Finally, we observed no obvious change for body weights during treatment and pathological examination from H&E staining images of major organs, indicating that the adminstration of SPN(NIR-3) induced little toxicity (Figure S20 and S21).

### Investigation of SPN(NIR-3)-mediated immunogenic cell death *in vivo*

To validate the mechanism of SPN(NIR-3)-mediated tumor therapy, the effects of photodynamic treatment on ICD production, DC maturation, and immune response activation were studied. Spleen of mice after treatments were collected for investigating the maturation of DCs using flow cytometry assay. The populations of matured DCs (CD80^+^CD86^+^) in treated group was positively related to the dose of SPN(NIR-3) (Figure 6G). Then, we revealed that SPN(NIR-3) with laser irradiation promoted the activation of T helper cells (CD4^+^) and cytotoxic T lymphocytes (CTLs) (CD8^+^) in mice spleens, compared with the control group (Figure 6H). In addition, the level of immune-relevant cytokines in spleen including interleukin-6 (IL-6) tumor and necrosis factor-α (TNF-α) were increased by 3.03- and 4.24-fold for the maximum dose of SPN(NIR-3) treated group compared to control group, respectively (Figure 6I). Furthermore, the translocation of CRT to the cell membrane surface was one of the hallmarks for ICD induced by photodynamic effect of SPN(NIR-3) *in vivo*, confirmed by tumor slices immunofluorescence staining of CRT (Figure 6J). Thus, SPN(NIR-3)-mediated photodynamic therapy could induce ICD of tumor, and thereby promote DC maturation and activate adaptive anti-tumor immune response, resulting in tumor growth inhibition (Figure 6A).

## Discussion

Currently, the relatively short-wavelength emission still limited the use of afterglow luminescence imaging for biological applications. In this study, by introducing pyrido pyrazine (PP) as a strong “acceptor”, we designed three kinds of “D-A” semiconducting polymers. Notably, the internal charge transfer between the “donor” and “acceptor” moieties led to the observed red-shift of wavelength. Among those three molecules, NIR-3 has the strongest near-infrared (NIR) fluorescence emission. And then NIR-1/NIR-2/NIR-3 were self-assembled to construct a novel NIR afterglow nano-reporter.

We selected SPN(NIR-3) as the subsequent probe because it has the strongest NIR afterglow emission and the reasons of it are as following: 1. NIR-3 has the largest number of thiophene structures in its main chain, which may provide more reaction sites for driving the afterglow luminescence. 2. ΔEst of NIR-3 is lower than that of NIR-1 and NIR-2, which makes NIR-3 more likely to undergo intersystem crossing (ISC) and then produced more ^1^O_2_. 3. NIR-3 has the strongest fluorescence emission, which makes it possible to generate the strongest afterglow signal. Particularly, such NIR afterglow luminescence emitted by SPN(NIR-3) was able to eliminate the effect of autofluorescence of tissues so as to enhance the signal to noise ratio and sensitivity, as compared to fluorescence.

At present, the unknown dose of ROS yeild during those ROS mediated cancer therapy (e.g. photodynamic therapy, radiotherapy, or chemodynamictherapy) may induce the unfavorable anti-tumor immunity 39, 52, 53. Becuase the effect of ROS on the immunogenic response may range from facilitation to inhibition, the monitoring of ROS level during treatment is essential for immunogenic cell death (ICD)-related immunotherapy 19, 54. Fortunately, the appropriate administration of SPN(NIR-3) and laser irradiation could result in the proper uplift of intracellular oxidative stress, leading to ICD, followed by the release of DAMPs from cancer cells to induce DCs maturation and upregulate cytokines (IL-6 and TNF-α) expression. Those process further activate effector T cells and ultimately enhance the host's antitumor immune response against tumors.

Since the NIR afterglow luminescence of SPN(NIR-3) was ^1^O_2_-dependent, the ^1^O_2_-related afterglow signal was closely correlated with the degree of photodyanmic effect, immunogenic cell death and cancer inhibition rate for cancer cells or solid tumor in mice. Thereby, this NIR afterglow imaging signal may be used as a metric for the initial stratification of ^1^O_2_-mediated immune responses and anti-cancer efficiency in the early stages of therapy, before tumor shrinking. Moreover, such ^1^O_2_ correlated afterglow luminescence can afford real-time information for adjusting various therapeutic parameters (such as dosage and irradiation time) during the process of cancer therapy, allowing for the individual therapy while avoiding the ineffective or excessive treatment.

Cancer hypoxia is unavoidable as a result of uncontrollable tumor cell proliferation and dysregulated development of tumor blood vessels 55. Furthermore, PDT-induced microvascular collapse would further impair O_2_ delivery and increase the hypoxic situation. The above-mentioned tumor hypoxia outcome would jeopardize the efficacy of afterglow luminescence and cancer treatment. To reverse the hypoxic condition of the tumor microenvironment, significant efforts may be made in the future to develop efficient strategies to refuel O_2_ in the tumor microenvironment to boost afterglow luminescence and PDT, such as delivering oxygen-saturated perfluorocarbons 56, using oxygen producing materials 57 or developing of type I photosensitizer with less oxygen requirement 58.

## Conclusion

In summary, we have developed pyrido pyrazine-based nanoparticles as a new type of NIR afterglow luminescence nanoprobe for monitoring immunotherapy. We extended the emission wavelength of semiconducting polymers *via* inducing the strong “acceptor” (pyrido pyrazine) and further modulated their afterglow luminescence *via* screening “donor”. Next, we assembled them into semiconducting polymer nanoparticles (SPN) that showed longer - wavelength emission (> 800 nm) and higher signal to noise ratios for afterglow imaging, in contrast to fluorescence imaging. Moreover, the as-prepared SPN was able to monitor ^1^O_2_ during laser irradiation, cancer inhibition rate and thereby immunogenic cell death in real time during ^1^O_2_-mediated cancer therapy itself *via* afterglow imaging. Thus, this afterglow system provided a facile and non-invasive method for predicting anti-cancer efficacy, which was critical for individual immunotherapy and minimizing toxicity.

## Materials and methods

### Synthesis of semiconducting polymers

In 50 mL two-necked flask, the monomer of M0 (0.20 g, 0.21 mmol) and the monomer M1, M2 or M3 were dissolved with 10 mL toluene were dissolved in 10 mL toluene, respectively. After the repeated pumping and argon filling for three times, the Pd(PPh_3_)_4_ (19 mg, 0.01 mmol) is quickly added into two-necked flask. Then, the reaction was heated to 110 °C and stirred for 24 hours. Next, the reaction solution was cooled to room temperature before poured into 200 mL methanol to precipitate the product. Subsequently, the solid of product were collected by filter paper and extracted with methanol, hexane, acetone and chloroform by Soxhlet extractor for 12 h each. The chloroform extracting solution was cooled to room temperature and concentrated by rotary evaporation. Finally, the product was precipitated in methanol, filtered and dried to obtain purple solids NIR-1, NIR-2, NIR-3. The specific synthetic ratio was shown in the following table.

### Synthesis of semiconducting polymer nanoparticles

First, a THF mixture solution was prepared by dissolving various semiconducting polymer (e.g., NIR-1, NIR-3, NIR-2, MEHPPV, PFODBT, 200 μg) and 5 mg DSPE-PEG into 1 mL THF by sonication. Second, the above solution was rapidly injected into 9 mL water under continuous sonication. After 10 min of sonication, THF was evaporated. The resulted nanoparticles were washed three times with water using 100 KD centrifugal filter tube and stored in the dark at 4 °C.

### Afterglow and fluorescence luminescent imaging in solution

Afterglow and fluorescence luminescence images were carried out using IVIS living animal imaging system in bioluminescence (no excitation) and fluorescence modes, respectively. For acquisition of afterglow luminescence images in solution, semiconducting polymer nanoparticles solution was placed into PCR tubes (500 μL), and irradiated with 660 nm laser for different times. Afterglow and fluorescence luminescence images were analyzed by ROI analysis using the Living Image 4.0 Software. The fluorescence imaging parameters: Fluorescence modes; Excitation: 635 ± 10 nm; Emission: ICG; Exposure time: 1 s; Field of view: C. The afterglow luminescence imaging parameters: Bioluminescence modes; Open filter; Exposure time: 30 s; Field of view: C.

For investigating afterglow intensities of SPN (NIR-1/NIR-2/NIR-3), three SPN (800 μg/mL of NIR-1/NIR-2/NIR-3) were pre-irradiated with 660 nm laser for 15 s (0.8 W/cm^2^), immediately followed by afterglow imaging. Exposure time: 10 s; Field of view: C.

For measurement of afterglow emission band, SPN(NIR-3) (400 μg/mL of NIR-3) were pre-irradiated with 660 nm laser for 15 s (0.8 W/cm^2^) and SPN(MEHPPV) or SPN(PFODBT) were irradiated with white light for 15 s (3 mW/cm^2^). The afterglow signals were acquired through four different channels (GFP: 510-570 nm; DsRed: 570-650 nm; Cy5.5: 690-770 nm; ICG: 820-880 nm), respectively. Exposure time: 30 s; Field of view: C.

For investigating afterglow intensity after cyclic irradiation, SPN(NIR-3) (800 μg/mL of NIR-3) were irradiated with 660 nm laser for 15 s (0.8 W/cm^2^), immediately followed by afterglow imaging.

For investigating afterglow decay time after irradiation, SPN(NIR-3) (800 μg/mL of NIR-3) were irradiated with 660 nm laser for 15 s, immediately followed by afterglow imaging.

For measuring the afterglow intensity of SPN(NIR-3) excited by heating, SPN(NIR-3) solution (200 μg/mL) was heated to 25 °C, 37 °C, 45 °C, 50 °C and 60 °C for 2 min, respectively, immediately followed by afterglow imaging.

For investigating whether the luminescence involving with ^1^O_2_ or not, SPN(NIR-3) (100 μg/mL) were incubated with PBS, Na_2_MoO_4_ (1 mM), H_2_O_2_ (2 mM), or Na_2_MoO_4_ (1 mM) + H_2_O_2_ (2 mM) for 30 s, immediately followed by afterglow imaging.

For investigating afterglow signals of SPN(NIR-3) under hypoxia condition, 50 μg/mL SPN(NIR-3) were sealed in a glass tube under nitrogen, then irradiated with 660 nm laser for 15 s, immediately followed by afterglow imaging.

For investigating the tissue-penetration of afterglow or fluorescence imaging, SPN(NIR-3) (800 μg/mL of NIR-3, 100 μL) were added into PCR tubes (500 μL) and irradiated with 660 nm laser for 25 s (0.8 W/cm^2^). Immediately after irradiation, those samples were covered with chicken tissue of varying thicknesses (0, 2, 6, 15, 20 mm) and the afterglow or fluorescence images were acquired. Exposure time: 30 s for afterglow, 1 s for fluorescence; Field of view: C.

### Singlet oxygen detection in solution

For SOSG testing, singlet oxygen sensor green (SOSG) was employed to evaluate the singlet oxygen generation by NIR-3 during laser irradiation. SPN(NIR-3) (2 μg/mL of NIR-3) were mixed with SOSG (final concentration of 3.34 μM) and irradiated with 660 nm laser (0.8 W/cm^2^) for a series of times. SPN(MEHPPV), SPN(PFODBT) irradiated with 5 mW/cm^2^ white light for 5 min. Then, the fluorescence intensity of SOSG was measured at 494 nm of excitation. The singlet oxygen generation was quantified by comparing the SOSG fluorescence at 530 nm.

For ESR testing, the trapping agent TEMP was employed to detect the generation of ^1^O_2_. 20 μL SPN(NIR-3) (2 mg/mL of NIR-3) and 20 μL TEMP (1 M) were mixed and then irradiated with 660 nm laser for 5 s (0.8 W/cm^2^) or not. Immediately after addition, ESR was conducted to measure ^1^O_2_ signal.

## Supplementary Material

Supplementary materials and methods, figures.

## Figures and Tables

**Scheme 1:**
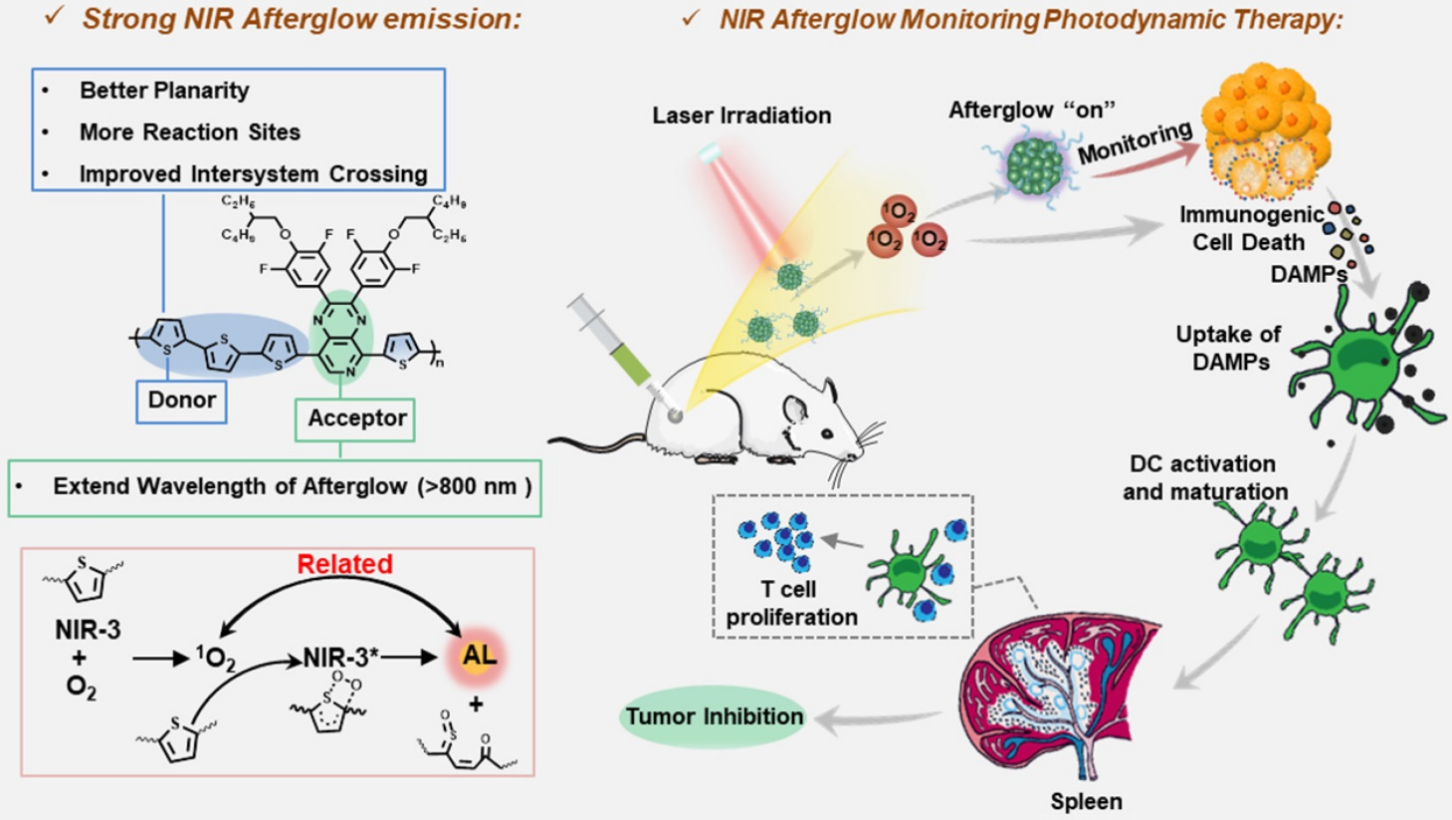
Schematic illustration of molecular structures and NIR afterglow imaging for monitoring cancer immunotherapy.

**Table 1:**
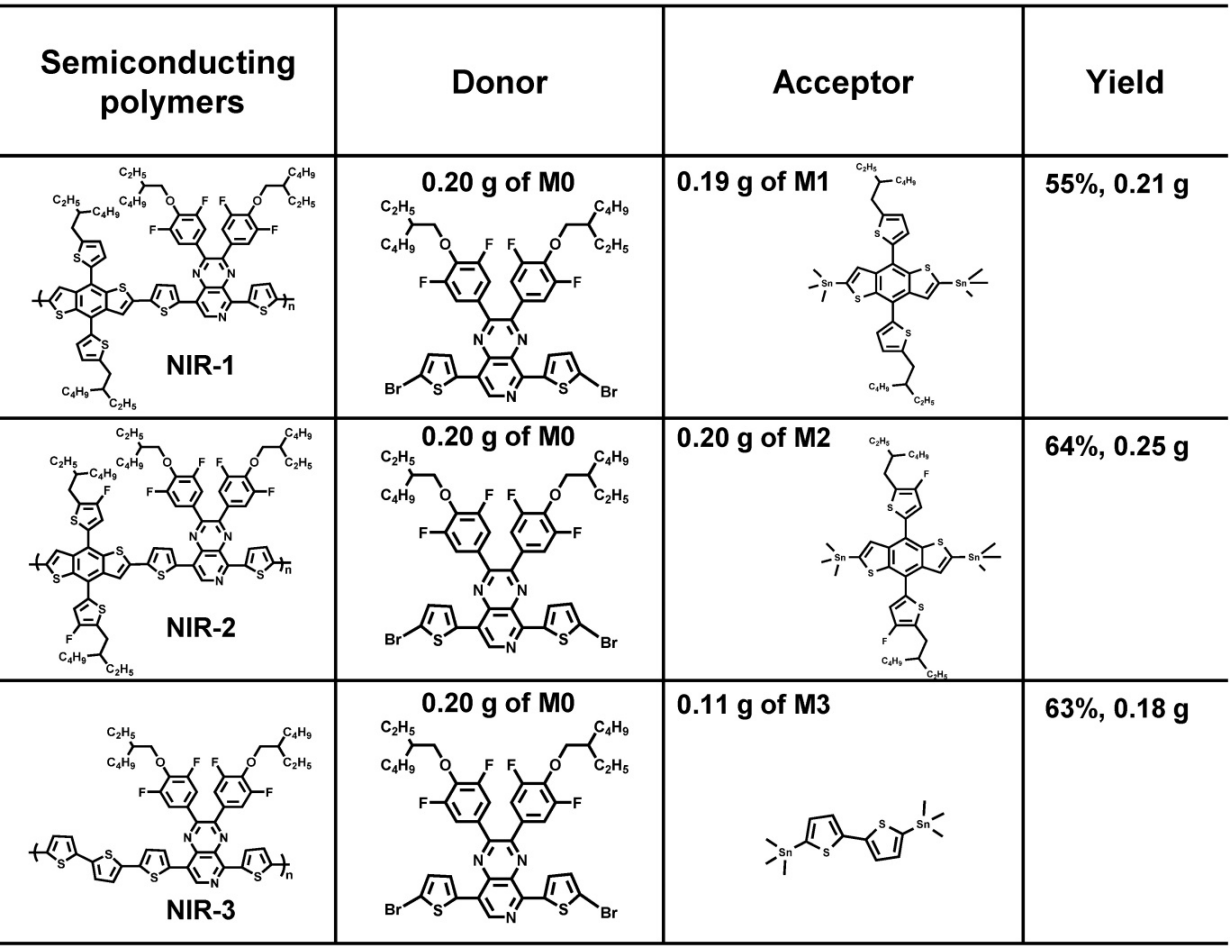
The specific synthetic ratio of three semiconducting polymers
