# Supplementary material for: A novel afterglow nanoreporter for monitoring cancer therapy
Source: Theranostics. 2022 Sep 25;12(16):6883–97. doi: 10.7150/thno.77457 (PMC9576607; doi:10.7150/thno.77457)
Supplement: Supplementary file 1 — Supplementary materials and methods, figures. [file thnov12p6883s1.pdf]

Supplementary Information (SI)

A novel afterglow nanoreporter for monitoring cancer therapy

Shiyi Liao[1,3], Youjuan Wang[1,3], Zhe Li[1], Ying Zhang[1], Xia Yin[1], Shuangyan Huan[1], Xiao-Bing Zhang[1], Sulai Liu[2,]* and Guosheng Song[1,]*

1. State Key Laboratory of Chemo/Bio-Sensing and Chemometrics, College of Chemistry and Chemical Engineering, Hunan University, Changsha 410082, P. R. China

2. Department of Hepatobiliary Surgery, Hunan Provincial People's Hospital/The First Affiliated Hospital of Hunan Normal University, Changsha, Hunan Province, People's Republic of China.

3. These authors contributed equally

* Corresponding author: E-mail: songgs@hnu.edu.cn (Guosheng Song), liusulai@hunnu.edu.cn (Sulai Liu).

Materials and Characterization.

NIR-1, NIR-2, NIR-3 was synthesized according to the literature [1]. Pluronic-F127 and singlet oxygen sensor green (SOSG) were purchased from Sigma-Aldrich. 2,2,6,6-Tetramethyl-4-piperidone (TEMP) and 2,2-dimethyl-3,4-dihydro-2H-pyrrole 1-oxide (DMPO) were obtained from Macklin. 1,2-dimyristoyl-sn-glycero-3-phosphoethanolamine-N-[methoxy (polyethylene glycol) (DSPE-PEG, Mw = 2000) was purchased from Shanghai TuYongBio Tech. Inc. Calreticulin (CRT) and high mobility group box 1 (HMGB1) antibodies were purchased from Cell Signaling Technology. ATP bioluminescent assay kit was purchased from Nanjing Jiancheng Bioengineering Institute. Mouse interleukin-6 (IL-6) ELISA Kit and mouse tumor necrosis factor-α (TNF-α) high sensitivity ELISA Kit were purchased from MultiSciences. All buffers and solutions were prepared using ultrapure water prepared through a Millipore Milli-Q water purification system (Millipore, USA).

Transmission electron microscope (TEM) images were performed by JEM-2100F (JEOL). Dynamic light scattering (DLS) was measured on Malvern Zetasizer Nano ZS90 (Malvern). The absorbance was recorded by ultraviolet-visible absorption spectrometry (UV-1800, Shimadzu) or microplate reader (SpectraMax iD3). The Fourier transform infrared (FTIR) spectra were recorded on an FTIR spectrometer (IR Affinity-1, Shimadzu, Japan). The fluorescent spectra were obtained by fluorescence spectrophotometer (F-7000, HITACHI). Fluorescence confocal images of live cells were performed through a Nikon A1plus multiphoton laser scanning confocal microscopy. The fluorescent and afterglow luminescent images of centrifuge tube or mice were obtained via an IVIS living animal imaging system (Caliper, U.S.A.) equipped with a cooled charge coupled device (CCD) camera. Flow cytometry assay was performed on a FACS Calibur flow cytometer (Becton Dickinson, USA).

Tissue-Penetration Depth of Fluorescence Imaging. For investigating the tissue-penetration of fluorescence imaging of SPN(NIR-3) and SPN(MEHPPV), the SPN (800 μg/mL of NIR-3 or MEHPPV, 50 μL) were added into 96-well plates. SPN(NIR-3) were irradiated with 660 nm laser

for 15 s (0.8 W/cm^2), and SPN(MEHPPV) were irradiated with white light for 15 s (3 mW/cm^2). Immediately after irradiation, those samples were covered with mimic tissue53 of varying thicknesses (0, 4, 8, 12, 20 mm) and the fluorescent images were acquired. Exposure time: 10 s; Field of view: C.

Cell Culture and Cellular Experiment. CT26 cancer cells were cultured in 1640 (Dulbecco's modified Eagle medium) (GIBCO) supplemented with 10 % FBS (fetal bovine serum) (GIBCO) and antibodies (10 U/mL penicillin and 10 mg/mL streptomycin) in a humidified environment at 37 ^oC which contains 5% CO_2 and 95% air.

For 3-(4,5-dimethylthiazol-2-yl)-2,5-diphenyltetrazolium bromide (MTT) cells assay, CT26 cancer cells were seeded in 96-well plates and incubated with fresh 1640. After 24 h incubation, the culture medium in 96-well plates was replaced by fresh medium containing SPN(NIR-3) (final concentration: 0, 25, 50, 100, 200, 400 μg/mL of NIR-3) and incubated for another 4 h. Then, those cells received 10 min irradiation of 660 nm laser (0.6 W/cm2) or not at 4 ^oC, and were further incubated for another 24 h. Finally, the relative cellular viability was tested by the standard MTT assay using microplate reader.

For fluorescence confocal imaging of $^1O_2$ generation, CT26 cancer cells pre-inoculated into 4-well plates and incubated with fresh 1640 containing SPN(NIR-3) (25 μg/mL of NIR-3) or not. After 4 h incubation, those treated cells were washed and irradiated by white light for 10 min. Next, those cells were stained with SOSG (10 μM) and Hochest (10 μM) for 30 min, respectively, and washed three times by PBS. Finally, those cells were imaged by confocal laser scanning microscope (Hochest: λex = 405 nm; SOSG: λex = 488 nm).

For measuring ATP secretion, CT26 cancer cells were seeded in 96-well plates and further incubated with SPN(NIR-3) (final concentration: 0, 25, 50, 100, 200 μg/mL of NIR-3) for 4 h. Then, the cells were washed and irradiated for 10 min, followed by another 4 h of incubation. Subsequently, the supernatants of cell culture mediums were collected from the plate, and then centrifuged using 13,000 rpm for 10 min. The concentration of secreted ATP in the supernatant

was quantified using an ATP bioluminescent assay kit (Nanjing Jiancheng Bioengineering Institute) by IVIS living animal imaging system according to the manufacturer's instruction.

For immunofluorescence staining of HMGB1, CT26 cancer cells were seeded in 12-well plates and further incubated with SPN(NIR-3) (400 μg/mL) or not. After 4 h incubation, the treated cells were washed and irradiated with 660 nm laser at 4 ^{o}C for 10 min, and further incubated for another 4 h. Next, the cells were fixed with 4% paraformaldehyde, and successively incubated with anti-HMGB1 primary antibody (1:200 dilution with 1×PBS) at 37 ^{o}C for 1 h, with Alexa Fluor 594-conjugated secondary antibody (1:250 dilution with 1×PBS) at 37 ^{o}C for 30 min. The nucleus was stained by Hochest for 15 min. The fluorescence images were taken under confocal laser scanning microscope (Hochest: λ_{ex} = 405 nm; HMGB1: λ_{ex} = 561 nm).

For immunofluorescence staining of CRT expression, CT26 cancer cell were seeded in 12-well plates and incubated with SPN(NIR-3) (400 μg/mL) or not for 4 h. Then, the treated cells were washed and irradiated with 660 nm laser at 4 ^{o}C for 10 min (0.8 W/cm^2), followed by incubation of 4 h. Next, the cells were fixed with 4% paraformaldehyde, and stained with anti-CRT primary antibody (1:200 dilution with 1×PBS) at 37 ^{o}C for 30 min, APC-conjugated secondary antibody (1:250 dilution with 1×PBS) at 37 ^{o}C for 30 min, Hochest for 15 min, in sequence. Finally, those cells were observed by confocal laser scanning microscope (Hochest: λ_{ex} = 405 nm; CRT: λ_{ex} = 637 nm).

For flow cytometry analysis of CRT expression, CT26 cancer cells were seeded in 12 well-plates and were incubated with various concentrations of SPN(NIR-3) (e.g., 0, 25, 50, 100, 200, 400 μg/mL of NIR-3) for 4 h. Then, those cells were washed and irradiated at 4 ^{o}C for 10 min, followed by 4 h incubation. Subsequently, the cells were collected and stained with anti-CRT primary antibody (1:200 dilution with 1×PBS) at 37 ^{o}C for 30 min, APC-conjugated secondary antibody (1:250 dilution with 1×PBS) at 37 ^{o}C for 30 min, and then with propidium iodide (PI) (Beyotime Biotechnology; 1:200 dilution with 1×Assay Buffer) in turn. Finally, the samples were

analyzed by flow cytometry, to identify cell surface CRT. The fluorescence intensity of stained cells was gated in PI-negative cells.

Cancer Imaging and Therapy In Vivo. All animal experiments were performed in compliance with the relevant laws and approved by the institutional Animal Care and Use Committee of Hunan University. Female balb/c mice were implanted subcutaneously with 50 μL of PBS containing CT26 cancer cells (2×10^6) to develop tumor model.

For afterglow luminescence imaging of cancer therapy, mice bearing subcutaneous CT26 tumors were i.t. injected with SPN(NIR-3) (0, 250, 500, 1000 μg/mL of NIR-3, 50 μL) (n=4). For acquiring images, the mice were pre-irradiated by 660 nm laser for 25 s (0.8 W/cm^2). Then, afterglow and fluorescence images were immediately captured. After imaging, those mice received another 8 min (6-minute intervals of every two minutes in case of the overheating of tumor area) of 660 nm laser radiation (0.8 W/cm^2) for cancer therapy. In separate experiment, five groups of subcutaneous CT26 tumor bearing mice received identical treatments as described above. The tumor size and mouse body weights were continuously recorded for 17 days. The tumor volumes were calculated as $V = a\times b^2/2$ (a and b: the longest and shortest diameter of tumor respectively).

For evaluating CRT expression within tumor, 12 h post treatment above, the representative tumors from each group were collected for cyro-section and sliced for immunofluorescence staining of CRT. Those tumor slices were stained with anti-CRT primary antibody at 37 ^{o}C for 30 min, with APC-conjugated secondary antibody at 37 ^{o}C for 30 min, and with DAPI for 15 min, successively. Finally, the tumor slices were observed by the confocal laser scanning microscope (DAPI: λ_{ex} = 405 nm; CRT: λ_{ex} = 637 nm).

In another separate experiment, representative mice were sacrificed on day 5 after the treatments and their spleens were collected to filter through a 70 μm filter to prepare a single cell suspension. For evaluation of DC maturation, the single cell suspension was stained with fluorophore-conjugated anti-CD11c, anti-CD80 and anti-CD86 antibodies (Biolegend) according

to the manufacture protocols. For evaluation of spleen T cell population, the collected single cell suspension was stained with fluorophore-conjugated anti-CD3, anti-CD4 and anti-CD8 antibodies (Biolegend) according to the manufacture protocols. CytExpe flow cytometer was used to analyze the stained cells. Supernatant samples during spleens minced were collected for cytokine detection (mouse interleukin-6 (IL-6) and tumor necrosis factor-α (TNF-α)) using ELISA kits according to the manufacturer's suffusion.

For hematoxylin and eosin (H&E) staining, 24 h post treatment in the separate experiment, the representative tumors were collected from each group and fixed in 4% paraformaldehyde for H&E staining using the standard protocols. Besides, main organs such as hearts, livers, spleens, lungs, and kidneys were collected from mice on the 17th day post treatment for H&E staining.

Supplementary

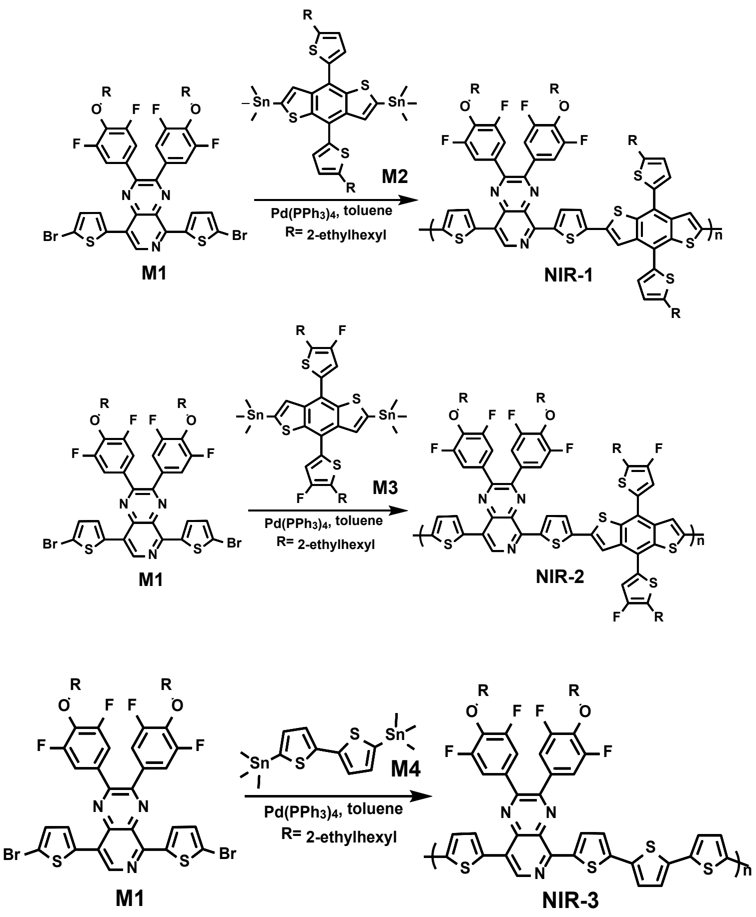

Figure S1. Synthesis route of three semiconducting polymers.

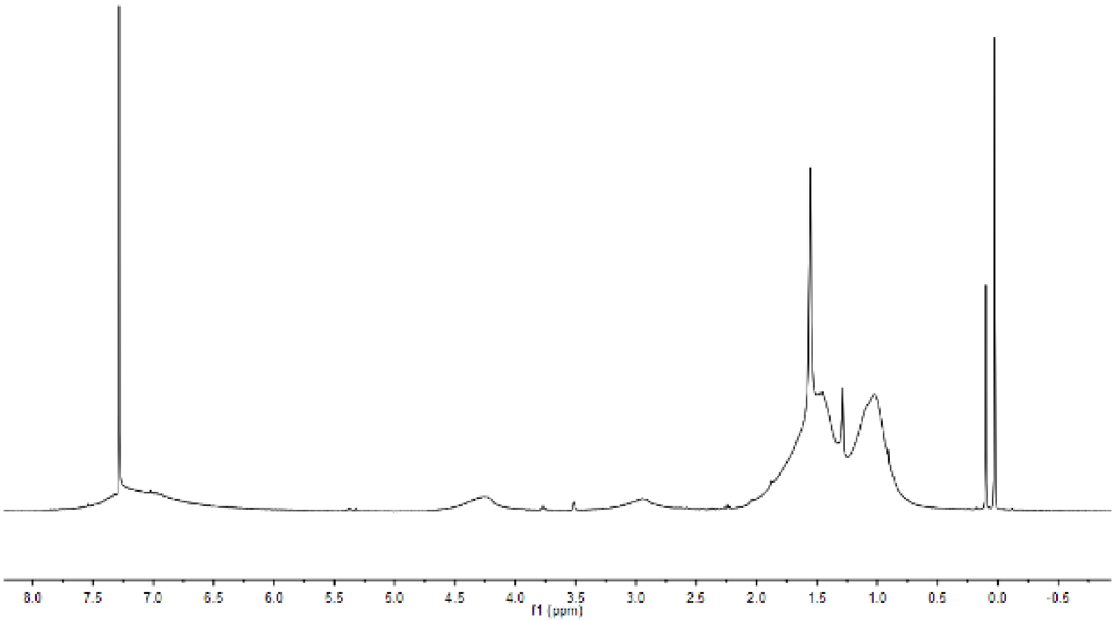

Figure S2. ^{1}H NMR spectrum of NIR-1.

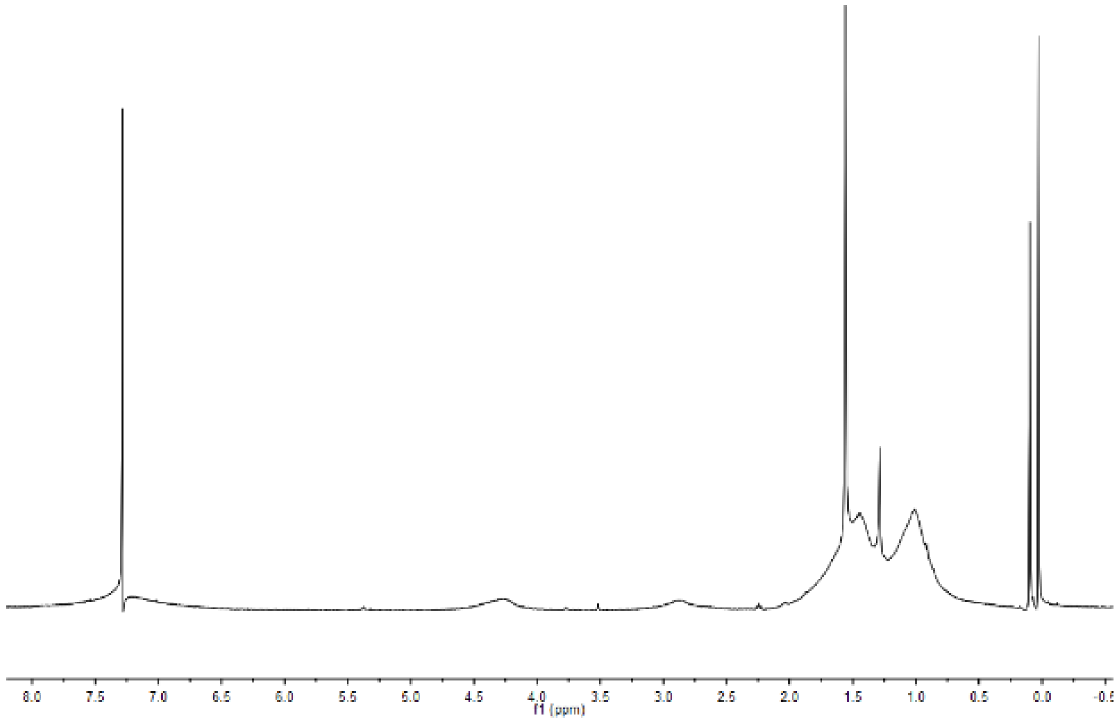

Figure S3. ^{1}H NMR spectrum of NIR-2.

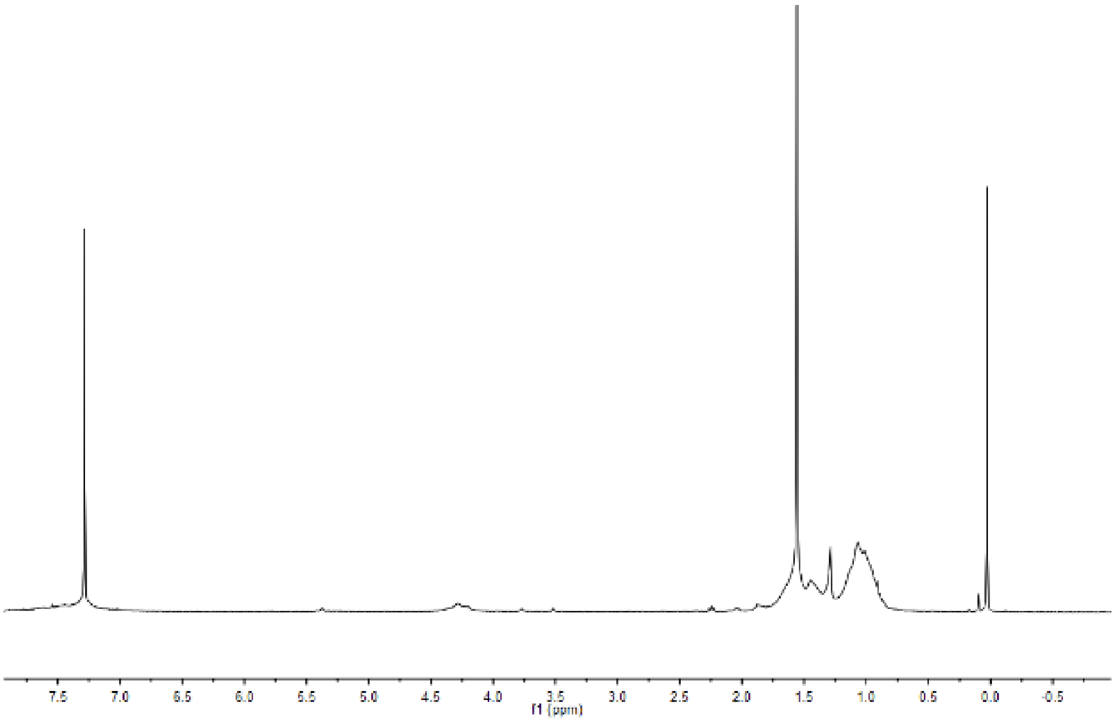

Figure S4. ^{1}H NMR spectrum of NIR-3.

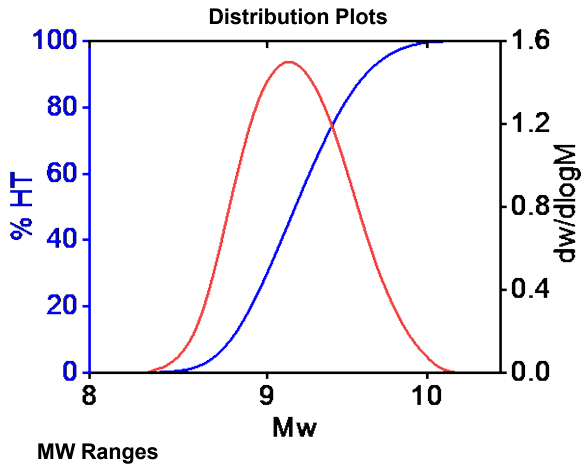

Figure S5. GPC measurement of NIR-3.

Parameters	NIR-1	NIR-2	NIR-3
Oscillator strength	2.176	2.3196	2.7031
ΔEst (nm)	1.7163	1.7449	1.5593

Figure S6. Oscillator strength and ΔEst of NIR-1, NIR-2 and NIR-3 through DFT calculation. Exchange functional: B3LYP. Basis sets: 6-31G*.

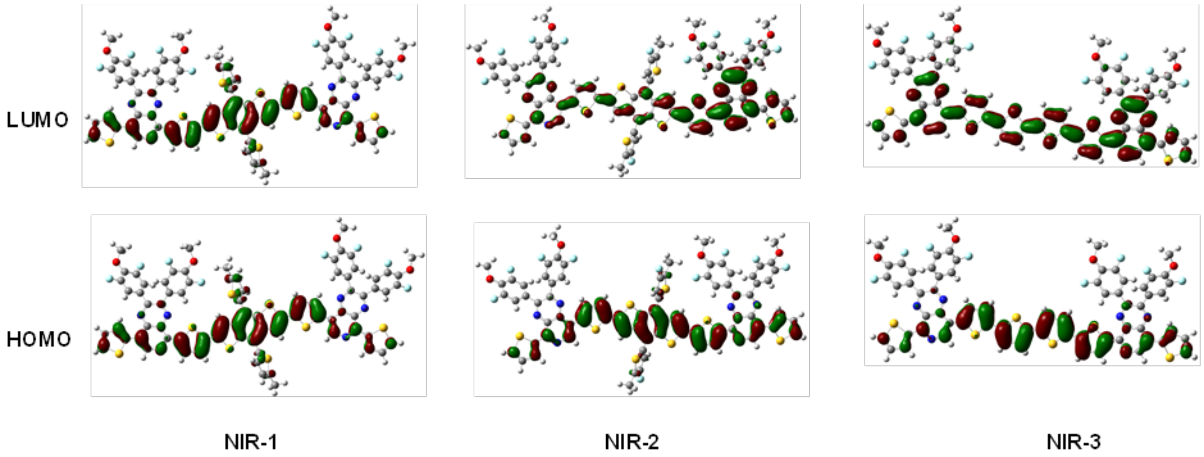

Figure S7. HOMO−LUMO distributions of NIR-1, NIR-2 and NIR-3. Exchange functional: B3LYP. Basis sets: 6-31G*.

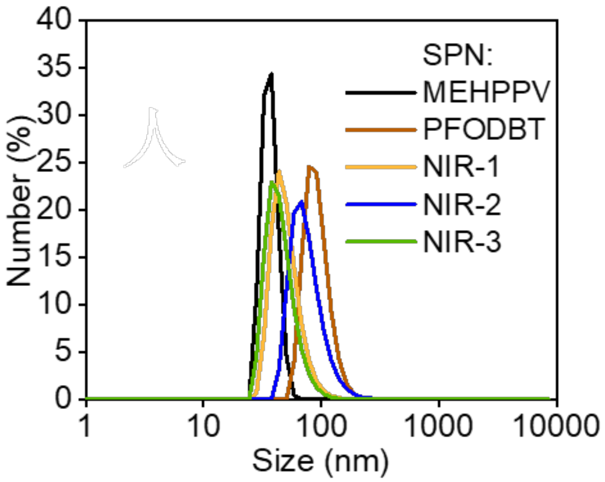

Figure S8. Dynamic light scattering size of various SPN in H_2O.

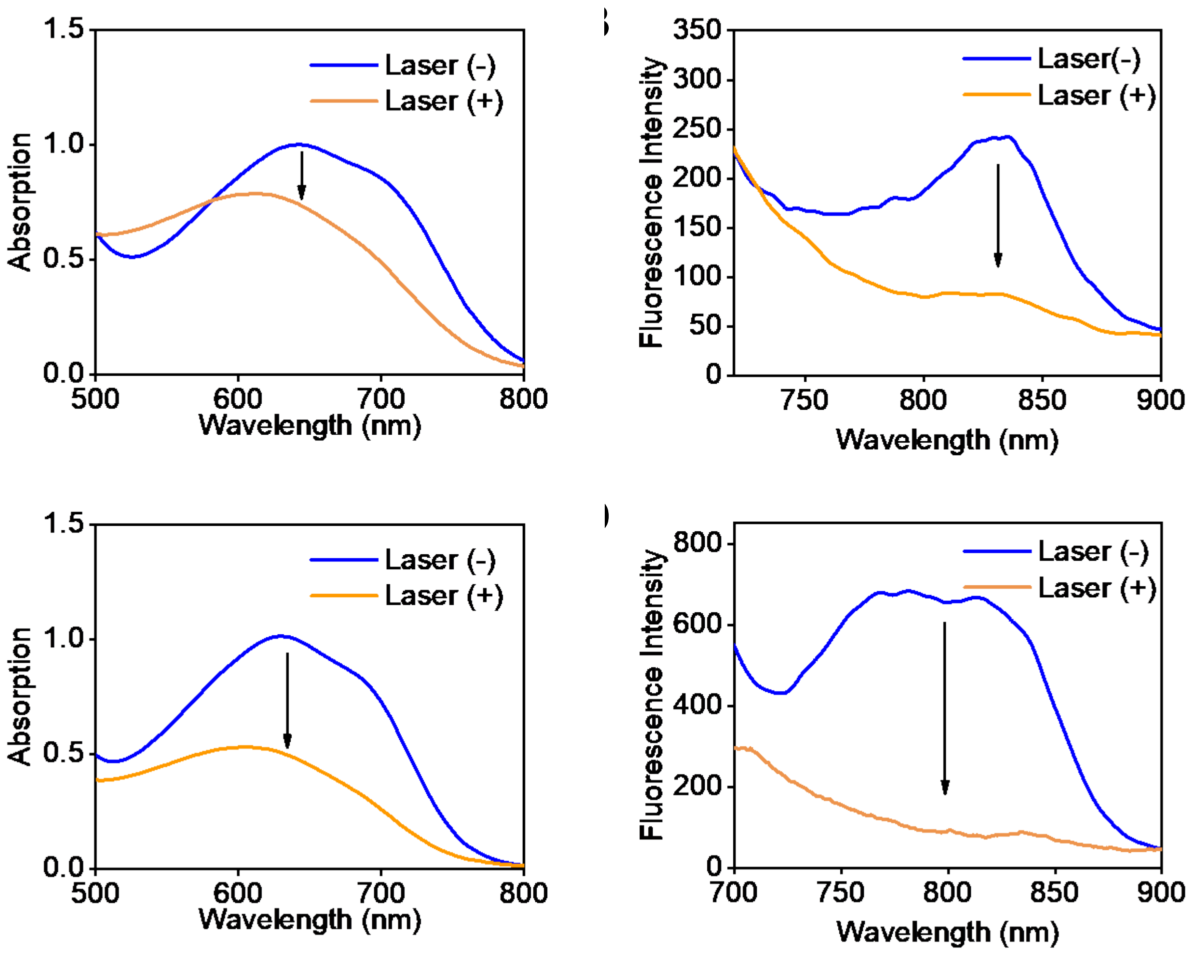

Figure S9. The absorption (A) and fluorescence (B) spectra of SPN(NIR-1) before and after irradiation. The absorption (C) and fluorescence (D) spectra of SPN(NIR-2) before and after irradiation.

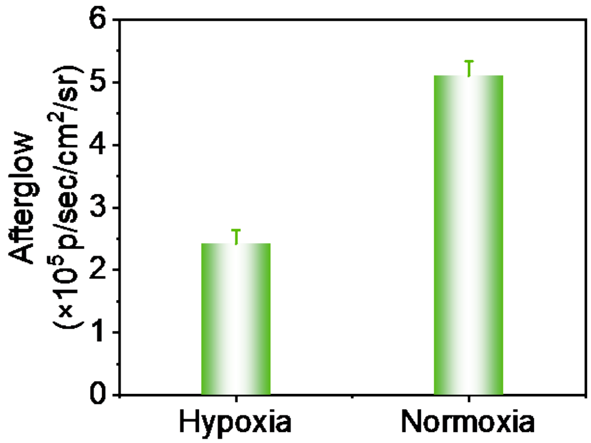

Figure S10. Afterglow intensities of SPN(NIR-3) under hypoxia or normoxia after 15 s irradiation (0.8W/cm^2).

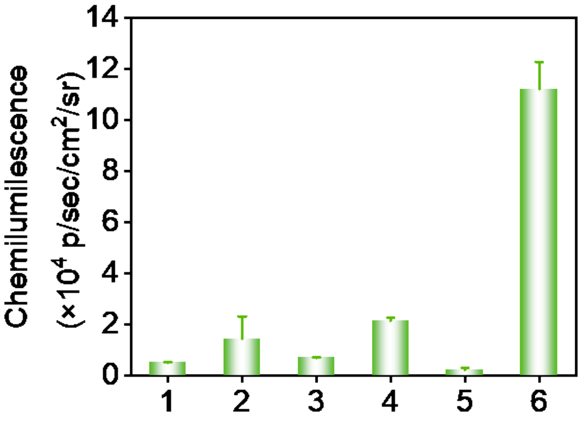

Figure S11. Luminescent intensity of SPN(NIR-3) incubated with different species (1: 10% DMSO, 2: 1 mM KO_2, 3: 200 μM Fe^{2+}, 4: 500 μM •OH, 5: 2 mM H_2O_2, 6: 1mM $^1O_2$).

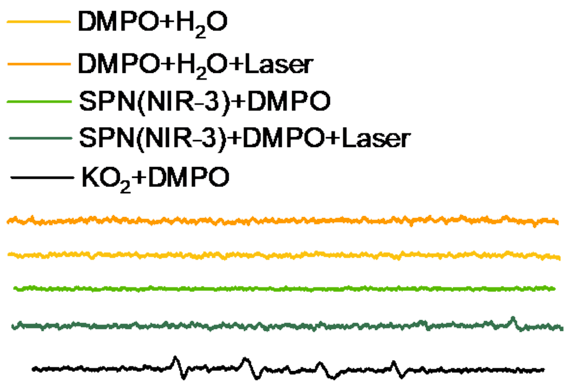

Figure S12. ESR spectra of DMPO + H_2O, DMPO + H_2O +Laser, SPN(NIR-3) + DMPO, SPN(NIR-3) + DMPO +Laser, KO_2 + DMPO.

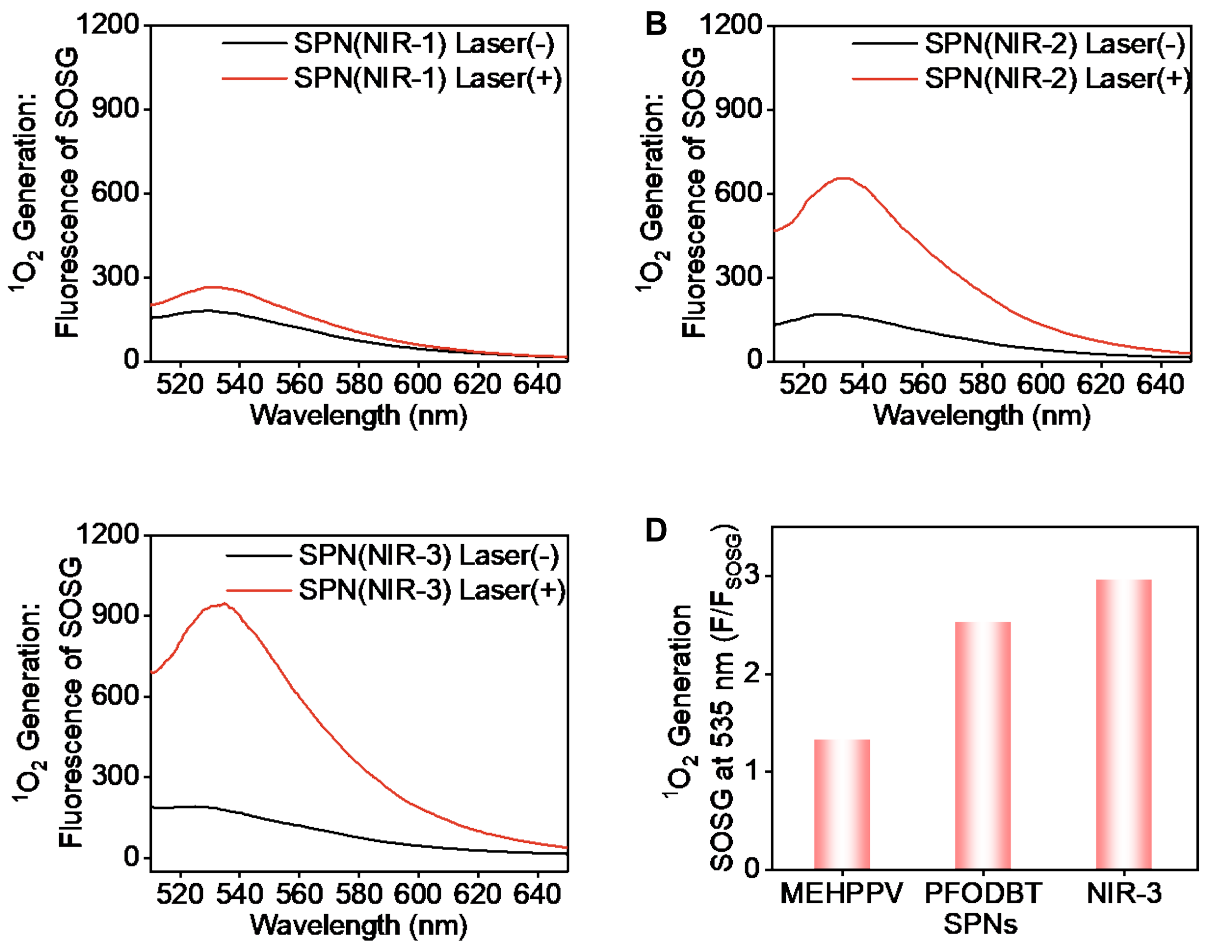

Figure S13. Fluorescence spectra of SOSG: $^1O_2$ generation capacity of various nanoparticles with laser irradiation (15 s) or not. 5 mW/cm^2 white light for SPN(MEHPPV) and SPN(PFODBT), 0.8 W/cm^2 660 nm laser for SPN(NIR-1/2/3).

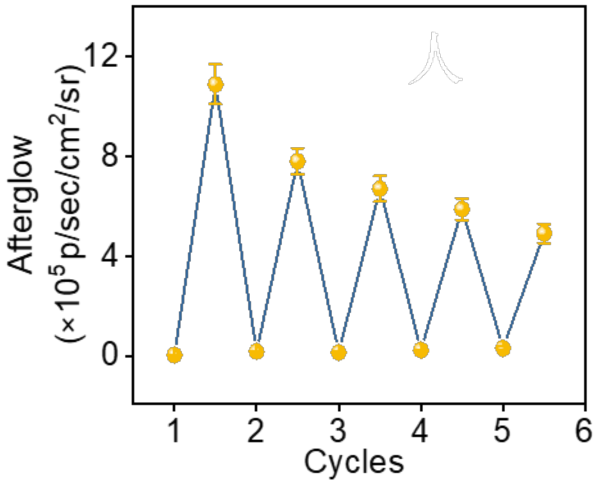

Figure S14. Afterglow emission intensities of SPN(NIR-3) as a function of light irradiation cycle.

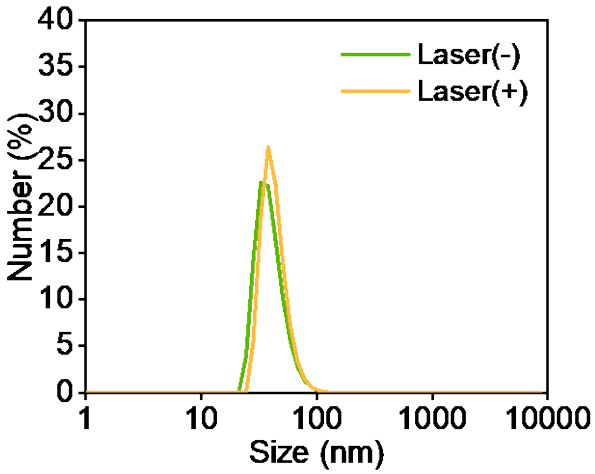

Figure S15. Dynamic light scattering of various SPN(NIR-3) with or without irradiation.

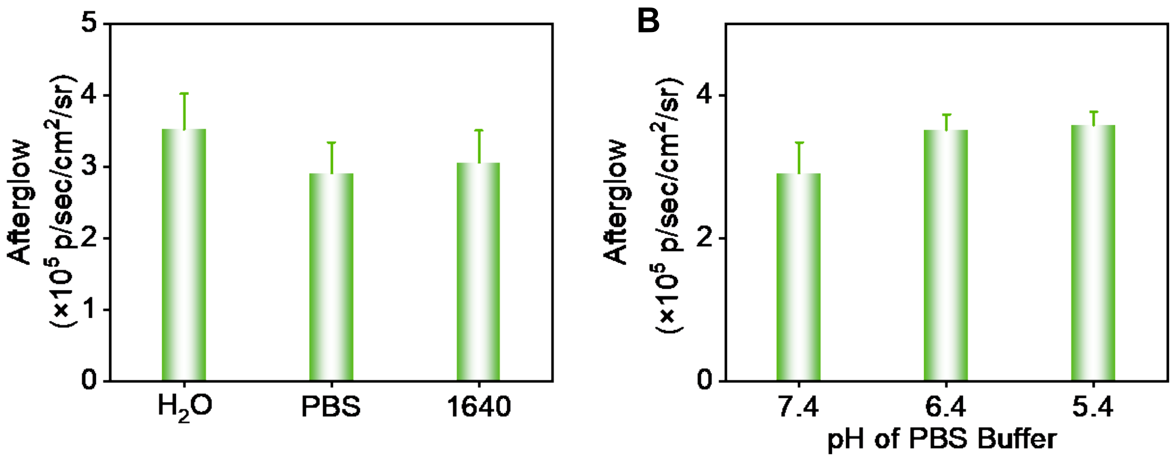

Figure S16. Afterglow intensities of SPN(NIR3) in different solutions.

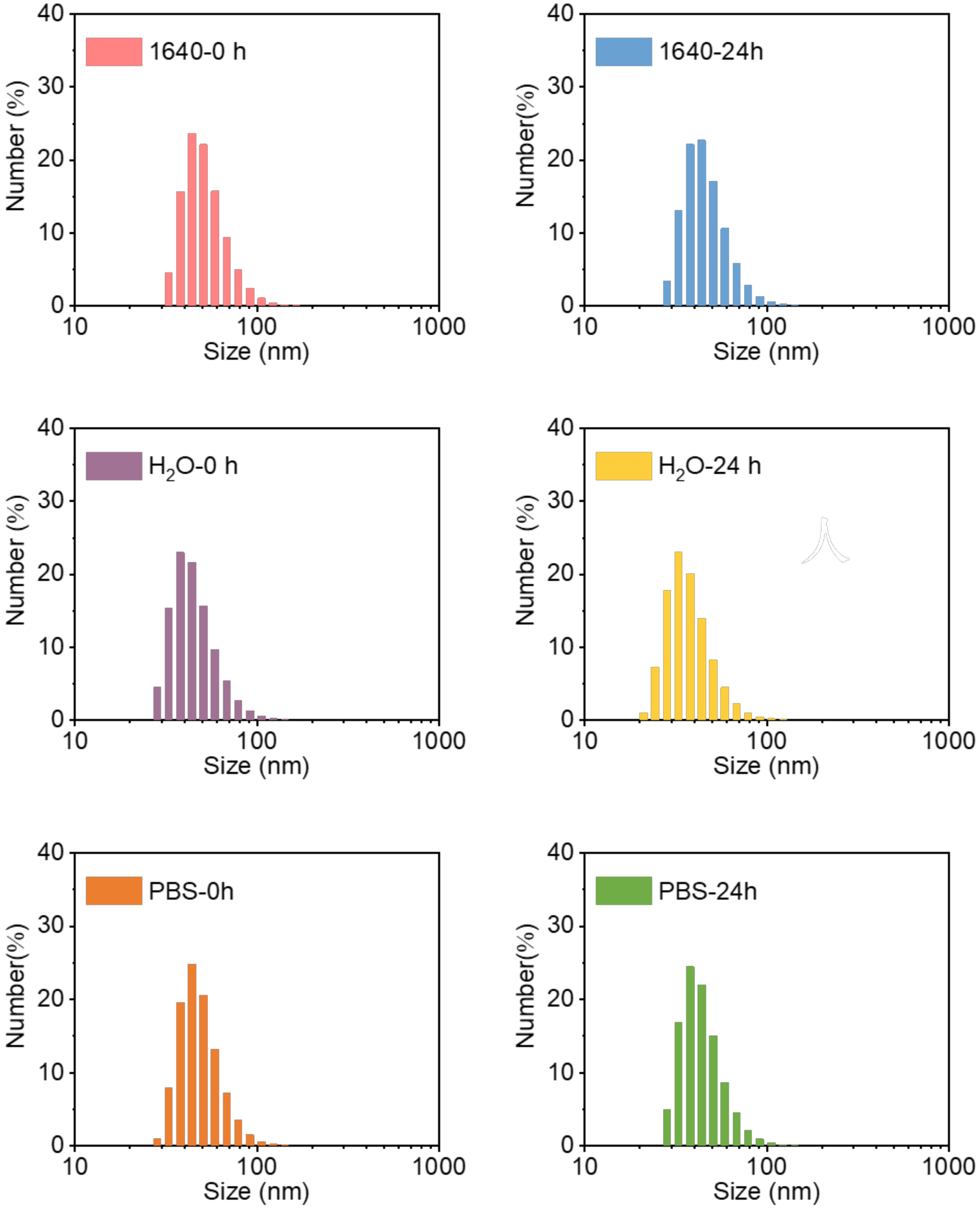

Figure S17. DLS sizes of SPN(NIR-3) in different solutions incubated for different times.

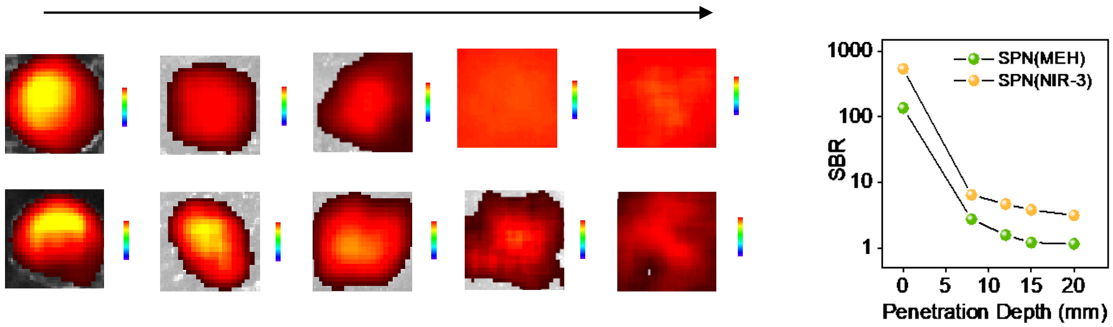

Figure S18. (A) Fluorescence images of SPN(NIR-3) and SPN(MEH) through mimic tissues of different thickness. **(B)** Signal-to-background ratio for fluorescence from (A).

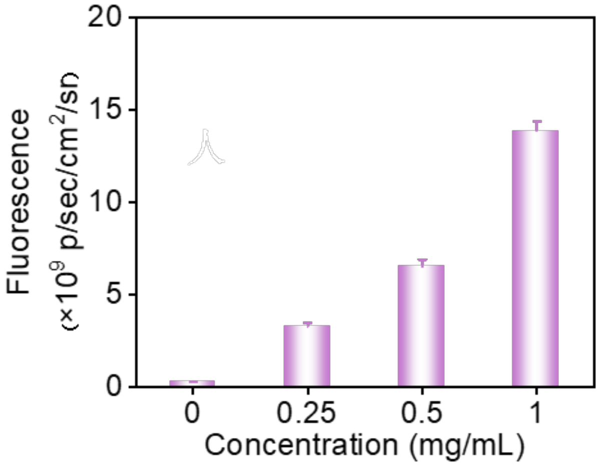

Figure S19. Corresponding quantification of fluorescence intensities for tumor areas in (6b).

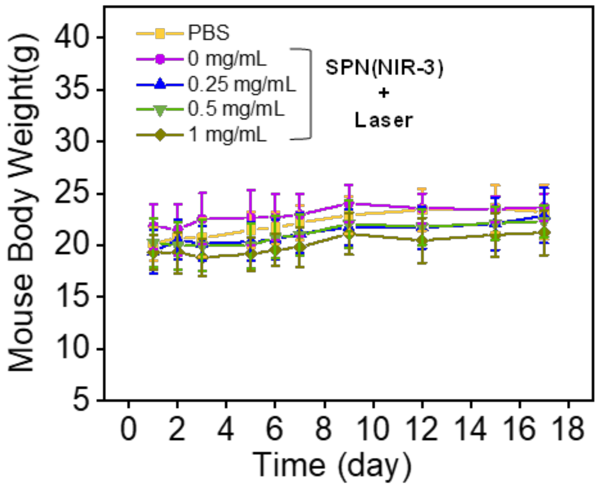

Figure S20. Mice body weights for each group during 17 day's observation after various treatments.

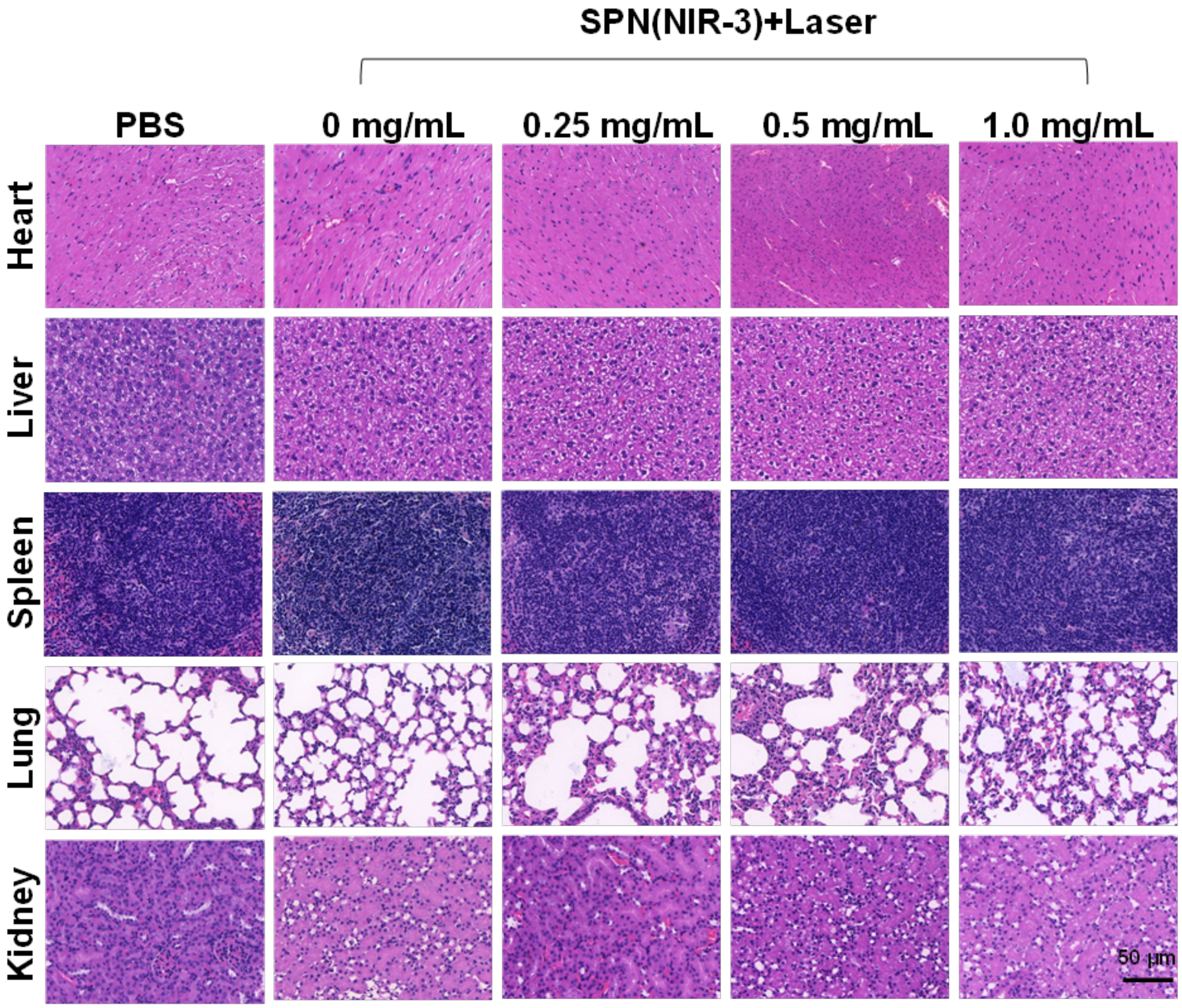

Figure S21. H&E staining images of major organs were collected from mice after various treatments.

REFERENCES

1. Wang T, Feng L, Yuan J, Jiang L, Deng W, Zhang Z-G, et al. Side-Chain Fluorination on The Pyrido[3,4-b]Pyrazine Unit Towards Efficient Photovoltaic Polymers. Sci China Chem. 2017; 61: 206-14.
